# Lipopolysaccharide from Crypt-Specific Core Microbiota Modulates the Colonic Epithelial Proliferation-to-Differentiation Balance

**DOI:** 10.1128/mBio.01680-17

**Published:** 2017-10-17

**Authors:** Tomoaki Naito, Céline Mulet, Cristina De Castro, Antonio Molinaro, Azadeh Saffarian, Giulia Nigro, Marion Bérard, Mélanie Clerc, Amy B. Pedersen, Philippe J. Sansonetti, Thierry Pédron

**Affiliations:** a Unité de Pathogénie Microbienne Moléculaire, INSERM U1202, Institut Pasteur, Paris, France; b Collège de France, Paris, France; c Yakult Central Institute, Tokyo, Japan; d Department of Agricultural Sciences, Porici (NA), Italy; e Dipartimento di Scienze Chimiche, Università di Napoli Federico II, Naples, Italy; f Animalerie Centrale, Institut Pasteur, Paris, France; g School of Biological Sciences & Centre for Immunity, Infection and Evolution (CIIE), University of Edinburgh, Edinburgh, United Kingdom; University of British Columbia

**Keywords:** homeostasis, intestinal stem cells, LPS, necroptosis

## Abstract

We identified a crypt-specific core microbiota (CSCM) dominated by strictly aerobic, nonfermentative bacteria in murine cecal and proximal colonic (PC) crypts and hypothesized that, among its possible functions, it may affect epithelial regeneration. In the present work, we isolated representative CSCM strains using selective media based upon our initial 16S rRNA-based molecular identification (i.e., *Acinetobacter*, *Delftia*, and *Stenotrophomonas*). Their tropism for the crypt was confirmed, and their influence on epithelial regeneration was demonstrated *in vivo* by monocolonization of germfree mice. We also showed that lipopolysaccharide (LPS), through its endotoxin activity, was the dominant bacterial agonist controlling proliferation. The relevant molecular mechanisms were analyzed using colonic crypt-derived organoids exposed to bacterial sonicates or highly purified LPS as agonists. We identified a Toll-like receptor 4 (TLR4)-dependent program affecting crypts at different stages of epithelial differentiation. LPS played a dual role: it repressed cell proliferation through RIPK3-mediated necroptosis of stem cells and cells of the transit-amplifying compartment and concurrently enhanced cell differentiation, particularly the goblet cell lineage.

## IMPORTANCE

The LPS from crypt-specific core microbiota controls intestinal epithelium proliferation through necroptosis of stem cells and enhances cell differentiation, mainly the goblet cell lineage.

## INTRODUCTION

The intestinal epithelium undergoes rapid renewal (i.e., 4 to 5 days). The Wnt/Notch pathway being essential to its regenerative capacity (1) that relies on stem cells located at the bottom of intestinal crypts. Intestinal stem cells maintain gut epithelial integrity by supporting epithelial proliferation followed by cell differentiation into the five intestinal lineages: Paneth cells, goblet cells, enteroendocrine cells, absorptive enterocytes, and tuft cells. In the colon, Paneth cells are not detected, at least according to the criteria used in the small intestine. One important feature of stem cells is their self-renewal capacity. Particular to the intestinal epithelium is its close contact with the microbiota and engagement in a symbiotic relationship with the commensal bacteria it contains (2). In order to determine the physiological implications of this symbiotic interaction, one needs to characterize the properties of the niche in which it occurs. Bacterial distribution is indeed a major parameter as illustrated, for instance, by the observations that *Alcaligenes* species are detected inside Peyer’s patches of the small intestine and isolated lymphoid follicles (3) and that the families *Lachnospiraceae* and *Ruminococcaceae* are enriched in the interfold region of the proximal colonic (PC) mucosa (4). In patients with inflammatory bowel diseases or irritable bowel syndrome, *Bacteroides*, clostridia, and *Escherichia coli* are enriched at the inflamed colonic mucosal surface (5–8). However, the complex relationship between microbial distribution and its influence on the host has not yet been elucidated.

We recently identified members of the *Acinetobacter*, *Stenotrophomonas*, and *Delftia* genera in the crypts of the murine cecum and proximal colon. We named this bacterial assemblage the “crypt-specific core microbiota” (CSCM) (9). On the basis of our recent results demonstrating a Nod2-mediated cytoprotective pathway of gut stem cells by bacterial muramyl-dipeptide (10), we decided to address the possible roles of CSCM members on crypt physiology and to characterize these strains and their possible functional impact.

Given the crypt localization of CSCM, we hypothesized that cells composing the crypt, including Lgr5^+^ stem cells located at the bottom of the crypt, cells of the transit-amplifying compartment, and upper noncycling and terminally differentiating epithelial cells, could be affected by CSCM. This cross talk may affect essential homeostatic parameters such as the death/proliferation balance and differentiation of colonic epithelial cells. We thus combined *in vivo* and *ex vivo* approaches, particularly three-dimensional (3D) culture models of organoids (11) to evaluate the regulatory roles of individual CSCM representatives on epithelial regenerative functions in the absence of involvement of other cell populations like stromal and immune cells that may also respond to microbes and indirectly affect the epithelium. We also wished to identify the most bioreactive bacterial components, anticipating that particular microbe-associated molecular patterns (MAMPs) may play a dominant role.

We showed here that CSCM representatives clearly affected the key parameters of epithelial regeneration. While proceeding from *in vivo* analysis to molecular and cellular analysis on organoids, we identified lipopolysaccharide (LPS) as the dominant agonist accounting for the changes observed *in vivo* in monocolonized gnotoxenic mice. Although under inflammatory conditions, circulating LPS levels are drastically elevated and appear to play a pivotal role (12), the impact of LPS at homeostasis, under healthy conditions, has not been analyzed in depth in the small intestine and not at all in the colon. It was shown that in mice deficient for Myd88, the adaptor molecule for Toll-like receptor (TLR) signaling, the proliferative state of the colonic crypt was higher than in wild-type mice (13), indicating a key role of TLRs through recognition of commensal bacteria in the regulation of intestinal epithelial regeneration and homeostasis. Moreover, according to some reports, in the small intestine, LPS either directly activates stem cells via TLR4, hence promoting epithelial proliferation, or triggers apoptosis (14). However, the role of LPS in colonic epithelial cells has not yet been fully investigated.

Given the importance of bacterial products, more specifically the endotoxin, and the crucial role of TLRs on intestinal homeostasis, we investigated the impact of commensal bacteria on the colonic epithelium *in vivo* and *in vitro* by monocolonization of germfree mice with CSCM members and by stimulation of colonic organoids with bacterial sonicates or purified LPS, respectively. Our results reveal a strong but dual role of LPS on intestinal epithelial regeneration combining a decrease in epithelial proliferative rate through a necroptotic pathway and an increase in epithelial differentiation.

## RESULTS

### Isolation and characterization of CSCM strains from murine intestinal crypts.

As we had identified, by pyrosequencing, members of the *Acinetobacter*, *Stenotrophomonas*, and *Delftia* genera in the murine colonic crypts (9), our first objective was to cultivate, isolate, identify, and characterize the CSCM strains. In order to obtain enrichment in bacteria resident in the colonic crypts, proximal colon tissues were washed with bleach to remove luminal bacteria. As *Acinetobacter* was the main bacterial genus recognized inside proximal murine crypts, we used a selective medium dedicated to *Acinetobacter* enrichment, while allowing the growth of other aerobic bacteria, combined with vigorous aeration. Bacteria isolated from proximal colonic tissues were identified by phenotypic characterization using the Biolog system, followed by Sanger sequencing of the 16S rRNA gene and two housekeeping genes (*rpoB* and *gyrB*). This allowed us to collect 10 strains of *Acinetobacter*, including 8 *Acinetobacter modestus* strains, 2 *Acinetobacter radioresistens* strains, 20 strains of *Delftia tsuruhatensis*, and 1 strain of *Stenotrophomonas maltophilia* among 40 preparations. We focused the present work on four representative species: *A. modestus*, *A. radioresistens*, *D. tsuruhatensis*, and *S. maltophilia*.

### CSCM members stably colonize germfree mice.

In order to confirm the crypt tropism of these four CSCM members following their isolation, we monocolonized germfree C57BL/6 mice by gavage with each of these four species. Colonization efficiency was followed by counting bacteria in the feces. CFU numbers indicated that the four species were able to colonize the intestinal tract throughout the time course of the experiment (see Fig. S1A in the supplemental material). Interestingly, following fluorescent *in situ* hybridization (FISH) analysis, bacteria were detected not only in the luminal space of the intestine but also deep in the colonic crypts, thus confirming the crypt tropism of these four species, whereas the crypts of the different segments of the small intestine (duodenum, jejunum, and ileum) remained devoid of bacteria, even in condition of monocontamination (Fig. S1B to F). In the case of *Acinetobacter*, this particular tropism was found in our previous study (9) by cohousing germfree mice with conventional mice. For a control, we colonized germfree mice with *Bacteroides fragilis* NCTC 9343, a member of the healthy intestinal microbiota that was not found in proximal colonic (PC) crypts in our previous metataxonomic studies in conventional mice. Fifteen days after monoassociation, *B. fragilis* colonized the whole intestine and was also retrieved in the proximal colonic crypts (Fig. S1G) as previously shown (15), indicating that monoassociation of germfree mice did not totally reflect the ecosystem of conventional mice. Moreover, this phenomenon was not limited to breeding mice housed in our controlled animal facilities and receiving standardized and sterilized chow. Indeed, in animals captured in the wild like wood mice and bank voles, colonic crypts showed a massive presence of *Acinetobacter* (Fig. S1H and I), demonstrating that the presence of this CSCM member did not reflect a breeding bias, and confirming its true tropism to the colonic crypts of several rodent species, particularly those living in wild conditions.

10.1128/mBio.01680-17.2 FIG S1  Colonization of germfree mice with CSCM members or with *B. fragilis*. (A) Number of CFU in the feces during colonization. (B to G) FISH analysis of proximal colonic tissue using a universal bacterial probe labeled with Alexa Fluor 555 (Eub338). Proximal colonic tissue from a germfree control mouse (B) or from mice monocolonized with *A. modestus* (C), *A. radioresistens* (D), *D. tsuruhatensis*, (E) *S. maltophilia* (F), and *B. fragilis* (G). Magnification, 630×. (H and I) FISH analysis of proximal colonic tissue from a wood mouse (magnification, ×200) (H) and bank vole (magnification, ×100) (I) using a probe targeting *Acinetobacter* 16S RNA labeled with Alexa Fluor 555. Bacteria (red) and DAPI stain (green) are indicated. Download FIG S1, JPG file, 0.2 MB. Copyright © 2017 Naito et al. 2017 Naito et al. This content is distributed under the terms of the Creative Commons Attribution 4.0 International license.

### CSCM members affect proliferative cells *in vivo.*

**FIG 1:**
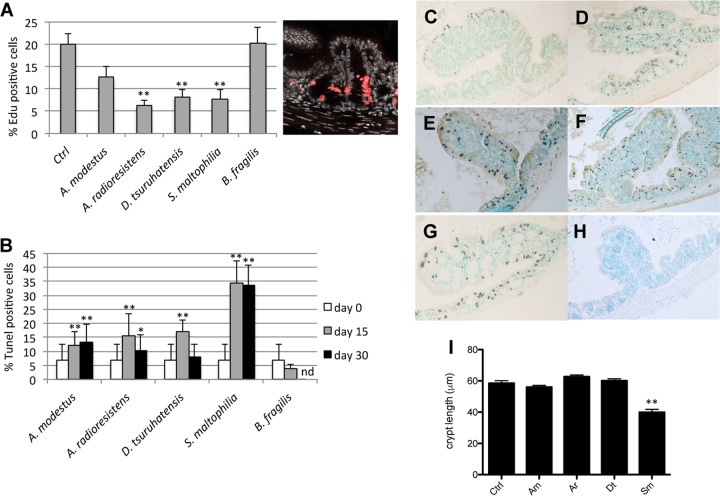
*In vivo* analysis of mice monoassociated with CSCM members. (A) Percentage of EdU^+^ cells in proximal colonic (PC) tissue from mice monocolonized with CSCM members or with *B. fragilis* on day 15 with a representative EdU staining of proximal colonic tissue from germfree mice as a control (Ctrl). (B) Percentage of apoptotic/dead cells in PC tissue from mice monocolonized with CSCM members on days 15 and 30 or monoassociated with *B. fragilis* on day 15. nd, not determined. (C to H) Representative pictures of apoptotic/dead cells of proximal colonic tissue from germfree mice (C) or from mice colonized for 15 days with *A. modestus* (D), *A. radioresistens* (E), *D. tsuruhatensis* (F), *S. maltophilia* (G), and *B. fragilis* (H). Magnification, 400×. (I) Proximal colonic crypt length of mice monocolonized with CSCM members *A. modestus* (Am), *A*. *radioresistens* (Ar), *D. tsuruhatensis* (Dt), and *S. maltophilia* (Sm). Data are expressed as means plus standard deviations (error bars). There were five mice in each group. Values that were significantly different from the value for the nonstimulated control group are indicated by asterisks as follows: *, *P* < 0.05; **, *P* < 0.01. Data from at least 50 crypts per section were examined for all histological parameters.

The effect of the selected strains on epithelial proliferation after 15 days of monocolonization was quantified using a mitotic index, based on 5-ethynyl-2′-deoxyuridine (EdU) incorporation. It indicated that three (of the four) strains induced a decrease in the mitotic index. Whereas *A. modestus* did not induce a significant decrease in the number of proliferative cells, the three other CSCM-associated species decreased the number of EdU-positive (EdU^+^) cells in the proximal colons of monocolonized mice (Fig. 1A). This decrease correlated with an increase in the number of dead cells as shown by the terminal deoxynucleotidyltransferase-mediated dUTP-biotin nick end (TUNEL) assay in Fig. 1B. A representative staining of dead cells is shown in Fig. 1C to G. Dead cells could be observed in the crypts of monocolonized mice after 15 days of colonization (Fig. 1D to G), whereas dead cells were sparsely observed in germfree mice (Fig. 1C). In mice monocolonized by *S. maltophilia*, we could indeed observe an increased number of dead cells by TUNEL staining analysis, and after 30 days of colonization, the percentage of dead cells remained high in the presence of this species, whereas it returned to near the level in germfree control mice after colonization with *A. radioresistens* and *D. tsuruhatensis* (Fig. 1B). Whereas EdU^+^ cells were localized in the lower half of the PC crypts (Fig. 1A), the increased number of TUNEL-positive (TUNEL^+^) cells was not restricted to a particular section of the colonic epithelium, such as the bottom of the crypts or the luminal surface of the colon, but were distributed throughout the epithelium. In this gnotoxenic context of monoassociation where *B. fragilis* was shown to be able to colonize proximal colonic crypts, we neither observed a decrease in the number of proliferative cells (Fig. 1A) nor an increase in the number of dead cells (Fig. 1B and H), indicating that even if allowed to dwell in crypts in the absence of an established microbiota, *B. fragilis* did not significantly affect the dynamics of epithelial regeneration. Moreover, the decrease of EdU^+^ cells and the increased number of dead cells are correlated with the decrease in the crypt length, mainly in mice colonized with *S. maltophilia* (Fig. 1I).

In order to determine whether the impact on stem cell and proliferative cell dynamics was restricted to the rather artificial situation of bacterial monoassociation of a gut that was not previously exposed to microbes, streptomycin-treated specific-pathogen-free (SPF) mice were colonized by gavage with a streptomycin-resistant isolate of *A. radioresisten*s. A total of 1 × 10^9^ streptomycin-resistant CFU/g of feces were recovered at days 5, 8, and 15 after colonization. Fifteen days after colonization, the percentages of proliferative cells in streptomycin-treated mice were 20.6 ± 2.4 and 12.2 ± 2.6 in streptomycin-treated mice colonized with the *A. radioresistens* streptomycin-resistant strain. In a similar trend, the percentage of TUNEL-positive cells increased from 1.65 ± 1.52 in control mice to 17.9 ± 5.4 in colonized mice, indicating that the effect of CSCM members was not restricted to germfree mice. In contrast, when streptomycin-treated SPF TLR4^−/−^ mice were colonized by gavage with the streptomycin-resistant isolate of *A. radioresistens* 15 days after colonization, the percentages of proliferative cells were 18.83 ± 6.84 in streptomycin-treated TLR4^−/−^ mice and 17.03 ± 4.25 in streptomycin-treated TLR4^−/−^ mice colonized with the *A. radioresistens* streptomycin-resistant strain. In the same way, the percentage of TUNEL-positive cells did not change from 3.33 ± 0.55 in TLR4^−/−^ mice to 3.63 ± 1.02 in colonized mice. These results indicate that in mice housed under conventional conditions, the colonization of the gut with a streptomycin-resistant CSCM strain induces an increase in TUNEL-positive cells and a decrease in proliferative cells in proximal colonic epithelium as observed in germfree associated mice and that the TLR4 pathway is involved in this phenomenon.

### Impact of CSCM strains on murine proximal colon organoids.

In order to analyze the effects of CSCM bacteria on intestinal epithelial cells, we developed an *ex vivo*, 3D culture model of proximal colon (PC) organoids (11). To establish that MAMPs and metabolites could interact with cells inside the organoid structure, we incubated crypts with a solution of fluorescein isothiocyanate (FITC)-dextran sulfate. As shown in Fig. S2A, FITC-dextran could be visualized inside the lumen of the organoid structure, indicating direct physical interaction between the added products and the cellular apex inside the organoid structure. On the basis of these results, PC purified crypts were incubated with or without filtered sonicated supernatants from each of the four CSCM species in order to analyze the global effect of the entire set of delivered MAMPs and bacterial products (i.e., proteins, stable metabolites) that potentially mediate the symbiotic cross talk. To confirm and quantify the effects of these bacterial preparations on epithelial physiology, three major parameters were considered: proliferation index, death index in the cell cycling compartment, and cell differentiation stage. Among the total set of organoids, we quantified two subgroups: “colonospheres” that showed spheric structure without appearance of surface protrusions and “colonoids” corresponding to differentiated organoids characterized by the presence of multilobulated structures due to the formation of stem cell-rich neocrypts on their surface after 2 days of culture (Fig. S2B).

10.1128/mBio.01680-17.3 FIG S2  PC organoid intake of FITC-dextran during organoid formation in matrigel. (A) Representative micrographs of FITC-dextran intake into PC organoids on days 3 and 4 are shown. PC crypts were cocultured with the optimal dose of FITC-dextran in matrigel, and collected PC organoids from matrigel were fixed with 4% paraformaldehyde (PFA) for 1 h and washed with PBS. Finally, organoids were observed by fluorescence microscopy. FITC-dextran (molecular weight of 4,000) was used as a probe for low-molecular-weight markers in sonicated CSCM isolates or for LPS. (B) Representative pictures of colonosphere and colonoid. Download FIG S2, JPG file, 0.1 MB. Copyright © 2017 Naito et al. 2017 Naito et al. This content is distributed under the terms of the Creative Commons Attribution 4.0 International license.

**FIG 2:**
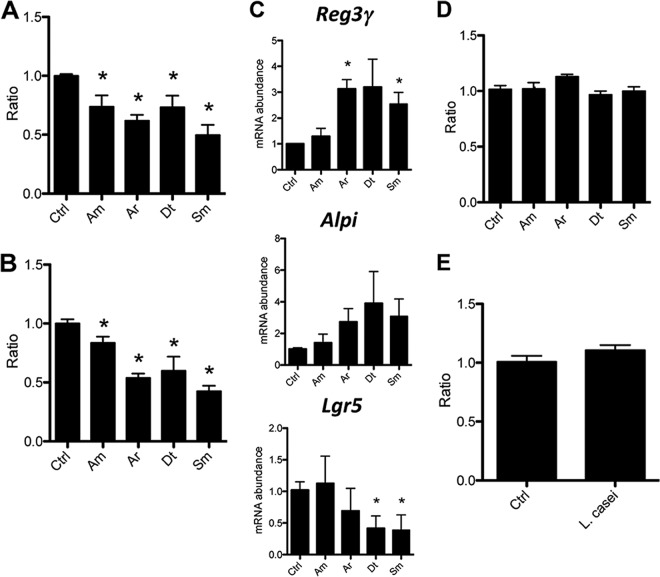
Effects of sonicated CSCM members on PC organoids. (A and B) Ratio of WT murine PC colonospheres (A) and colonoids (B) stimulated with sonicated CSCM isolates on day 7. The CSCM members are *A. modestus* (Am), *A*. *radioresistens* (Ar), *D. tsuruhatensis* (Dt), and *S. maltophilia* (Sm). There were four mice in each group. The ratio was calculated by normalizing the values (numbers) for stimulated organoids by the number of organoids without stimulation as a control (Ctrl). (C) Real-time PCR (RT-PCR) showing the gene expression in crypt cultures stimulated with sonicated CSCM isolates. (D) Ratio of total organoids from TLR4^−/−^ crypts stimulated with sonicated CSCM isolates. (E) Ratio of total organoids from WT crypts during stimulation with a sonicate from *Lactobacillus casei* ATCC 334. Data are expressed as means plus standard deviations (error bars). There were three mice in each group. Values that were significantly different (*P* < 0.05) from the value for the nonstimulated control group are indicated by an asterisk.

As shown in Fig. 2, all individual bacterial preparations induced a decrease in the respective ratio of colonospheres (Fig. 2A) and colonoids (Fig. 2B) compared to unstimulated controls 7 days after initiation of crypt cultures. The *S. maltophilia* preparations strongly decreased the ratio of colonospheres and colonoids compared to *A. modestus* sonicates, whereas *A. radioresistens* and *D. tsuruhatensis* sonicates induced an intermediate effect. Interestingly, whereas no significant differences were observed between the species in the decrease in the ratio of colonospheres, a significant difference in the ratio of colonoids was observed between *S. maltophilia* and *A. radioresistens* in comparison to *A. modestus*. Moreover, the *S. maltophilia* samples induced increased expression of *Reg3*γ and alkaline phosphatase (*Alpi*), a signature of epithelial cell maturation and differentiation, whereas the expression of *Lgr5*, a stem cell-specific marker, was strongly decreased by more than fourfold compared to nonstimulated samples (Fig. 2C). Similar gene expression profiles were observed when crypt cultures were stimulated with sonicates from *A. radioresistens* and *D. tsuruhatensis*, whereas *A. modestus* samples did not significantly affect expression of this set of genes (Fig. 2C).

We hypothesized that MAMPs were primarily involved in the observed modulation of epithelial development, particularly LPS, as suggested by the results obtained during colonization of streptomycin-treated TLR4^−/−^ SPF mice with the *A. radioresistens* streptomycin-resistant strain. We thus exposed crypts isolated from TLR4^−/−^ mice to the same extracts obtained from CSCM-associated species. Using similar experimental conditions and time frame, we did not observe any difference between stimulated and unstimulated crypt cultures. This strikingly indicated that among possible bacterial effectors, LPS and more specifically its endotoxin moiety, was the major molecular agonist altering organoid maturation patterns (Fig. 2D). As a matter of fact, a filtered sonicate of *Lactobacillus casei* ATCC 334, a Gram-positive species devoid of LPS, did not modulate the growth of organoids obtained from C57BL/6 proximal colonic crypts, providing strong complementary evidence that LPS is the dominant agonist of the cross talk in the colonic epithelium (Fig. 2E). Moreover, cell labeling showed that TLR4, the LPS receptor, was expressed on the apical side of epithelial cells in PC crypts (Fig. S3).

10.1128/mBio.01680-17.4 FIG S3  TLR4 is expressed on PC epithelial cells. Fluorescence micrographs showing immunostaining for TLR4 (red) in PC crypts from C57BL/6 and TLR4^−/−^ mice. Nuclei were stained with DAPI (green). Magnification, ×200. Download FIG S3, JPG file, 0.3 MB. Copyright © 2017 Naito et al. 2017 Naito et al. This content is distributed under the terms of the Creative Commons Attribution 4.0 International license.

### Purification of LPS and extraction and analysis of lipid A from CSCM-associated species.

**FIG 3:**
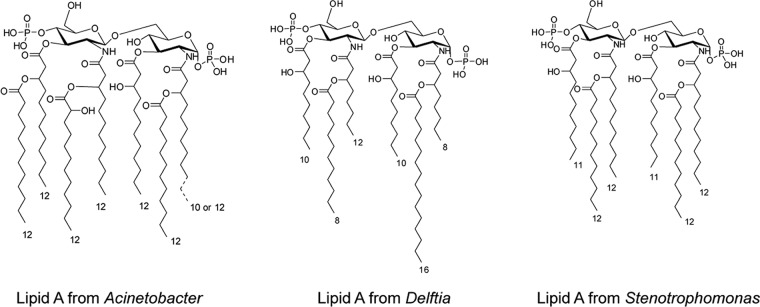
Structures of lipid A from *Acinetobacter*, *Delftia*, and *Stenotrophomonas* LPSs. The *A. modestus* and *A. radioresistens* lipid A differ by the presence of a C_10_ or C_12_ secondary fatty acid, respectively. The *S. maltophilia* lipid A is a blend of several species in which all the fatty acids can differ by one carbon atom and range from C_10_ to C_12_. The respective MALDI MS spectra are shown in the supplemental material (Fig. S4).

On the basis of the above results indicating a central role for LPS in the control of epithelial regeneration, we purified LPS from the four selected species and extracted and analyzed the respective lipid A moieties in order to decipher at the molecular level the signaling pathways involved. All four bacterial species were cultivated in large amounts, and LPS was extracted by a conventional procedure (16) followed by a further purification step encompassing a combination of enzymatic and chromatographic methods (17, 18). Lipid A, the bioactive moiety, was recovered from purified LPS by acid treatment before analysis by a combination of analytical biochemistry and matrix-assisted laser desorption ionization (MALDI) mass spectrometry (MS). The two *Acinetobacter* species were shown to possess a similar lipid A combining hexa- and hepta-acylated species. Conversely, *Delftia* lipid A appeared strongly underacylated with very short fatty acid chains. *Stenotrophomonas* lipid A appeared hexa-acylated with highly heterogeneous fatty acid substitutions, ranging between 10 and 15 carbon atoms (Fig. 3 and S4). The full chemical characterization will be described elsewhere.

10.1128/mBio.01680-17.5 FIG S4  MALDI MS spectra of lipid A from LPSs from *A. modestus* (A), *D. tsuruhatensis* (B) and *S. maltophilia* (C). The MS for *A. radioresistens* lipid A differs by the presence of a C_10_ or C_12_ secondary fatty acid. The MS spectrometry and determination of the whole structure will be reported elsewhere. Briefly, in all cases, the purified LPS underwent acetic acid hydrolysis, and the lipid A sediment was analyzed by MALDI MS, and where necessary, some peaks were identified by MS done in tandem (MS/MS) (where indicated in the figure). *S. maltophilia* lipid A was a mixture of several species differing by the length of fatty acids ranging from C_10_ to C_12_. Fatty acid analyses were conducted by the methods of De Castro et al. (17). Download FIG S4, JPG file, 0.2 MB. Copyright © 2017 Naito et al. 2017 Naito et al. This content is distributed under the terms of the Creative Commons Attribution 4.0 International license.

### Confirmation of LPS-mediated alteration of organoid maturation using purified LPS.

**FIG 4:**
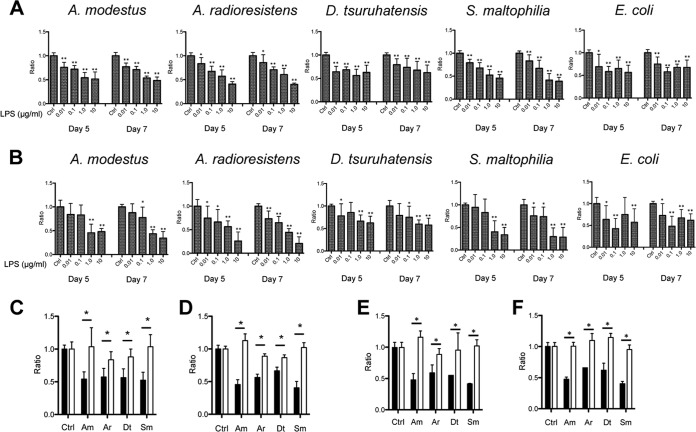
Effects of the LPSs purified from CSCM members on PC organoids. (A and B) Ratio of viable PC colonospheres (A) and colonoids (B) derived from WT crypts were calculated on days 5 and 7. PC organoids were stimulated with different concentrations (0.01 to 10 μg/ml) of LPS. (C and D) Ratio of colonospheres (C) and colonoids (D) during stimulation of organoids derived from WT mice (black bars) and TLR4^−/−^ mice (white bars) with LPS purified from CSCM members (1.0 μg/ml). (E and F) Ratio of colonospheres (E) and colonoids (F) during stimulation of organoids derived from WT mice with LPS (black bars) or with KOH-treated LPS (white bars) (1.0 μg/ml). The CSCM members are *A. modestus* (Am), *A. radioresistens* (Ar), *D. tsuruhatensis* (Dt), and *S. maltophilia* (Sm). There were six mice in each group. Values that were significantly different from the value for the nonstimulated control (Ctrl) group are indicated by asterisks as follows: *, *P* < 0.05; **, *P* < 0.01.

In order to confirm definitely that alteration in organoid maturation observed with bacterial sonicates was LPS dependent, we exposed PC organoids to increasing concentrations of purified LPS and calculated the ratio of colonospheres and colonoids at days 5 and 7 to those of control crypt cultures. As observed with filtered bacterial sonicates, pure LPS extracted from the two *Acinetobacter* species and from *S. maltophilia* induced a dose-dependent decrease in the ratio of PC colonospheres (Fig. 4A) and PC colonoids (Fig. 4B). This effect was also observed, although at a lower level, with a commercial *Escherichia coli* LPS used as control agonist, possibly due to lower degree of purification. Interestingly, pure *D. tsuruhatensis* LPS affected the colonosphere and colonoid ratios to a lesser extent than the other LPSs. This low agonistic signaling on TLR4 was likely due to a low acylation level compared to the other lipid A’s (19, 20). We determined the optimal concentration of LPS from CSCM members as 1.0 μg/ml for the next experiments. To confirm that this effect was dependent on TLR4, we analyzed the ratios of PC colonospheres and colonoids derived from TLR4^−/−^ mice. As shown in Fig. 4C and D, in agreement with the previously observed effect of bacterial filtered sonicates, the set of purified LPSs from CSCM members did not cause a decrease in the ratios of colonospheres and colonoids. In order to confirm these data, we analyzed the ratios of PC colonospheres and colonoids derived from wild-type (WT) mice after stimulation with KOH-treated LPS. This treatment deacylates lipid A, resulting in its detoxification. As shown in Fig. 4E and F, detoxified LPS did not decrease the colonosphere-to-colonoid ratio. Collectively, these results indicated that LPS from CSCM species altered the maturation of PC organoids, either by inhibiting their growth or by inducing their death through a TLR4-dependent pathway.

### LPS induces a decrease in the number of proliferative cells in PC organoids.

**FIG 5:**
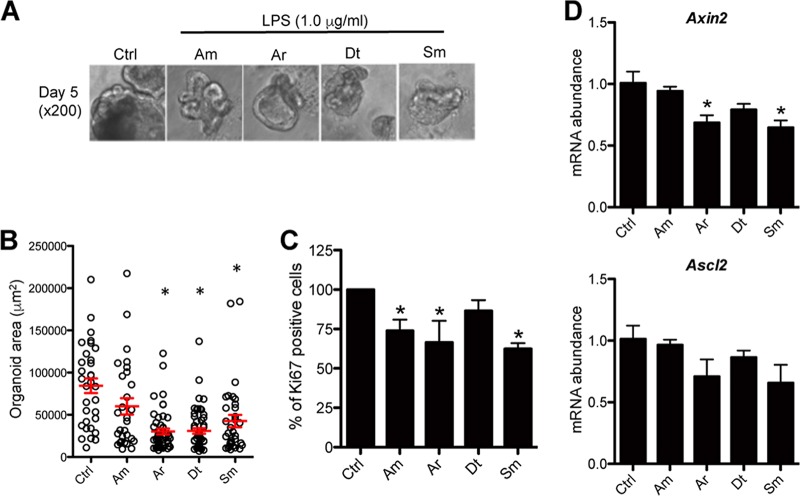
Effects of the LPS purified from CSCM members on size and transit-amplifying (TA) cells of PC organoids. (A) Representative bright-field micrographs of colonoids grown in matrigel for 5 days with LPS from CSCM members (1.0 μg/ml). The CSCM members are *A. modestus* (Am), *A. radioresistens* (Ar), *D. tsuruhatensis* (Dt), and *S. maltophilia* (Sm). (B) Individual area of day 5 PC organoid stimulated with LPS (1.0 μg/ml) in randomly selected fields. Red bars indicate averaged area of PC organoid. Values are means ± SE. *n* = 32 in non stimuli group, *n* = 29 in *A. modestus* group, *n* = 50 in *A. radioresistens* group, *n* = 54 in *D. tsuruhatensis* group, and *n* = 33 in *S. maltophilia* group as indicated. (C) Percentage of Ki-67^+^ TA cells in day 5 PC organoid stimulated with LPS. There were eight mice in each group. Values that were significantly different (*P* < 0.05) from the value for the nonstimulated control group are indicated by an asterisk. (D) RT-PCR showing *Axin2* and *Ascl2* mRNA abundances in WT PC organoids stimulated with LPS (1.0 μg/ml). Data are expressed as means plus standard deviations. There were three mice in each group. Values that were significantly different (*P* < 0.05) from the value for the nonstimulated control group are indicated by an asterisk.

The above results raised the possibility that LPS stimulation impaired the maturation of PC organoids. To evaluate this possibility, we measured the projected surfaces of PC organoids under bright-field microscopy and observed that LPS-stimulated PC organoids were significantly smaller than their unstimulated controls (Fig. 5A and B). The smaller size of organoids stimulated with LPS could be due to stem cells being targets of endotoxin-mediated signaling, thus accounting for decreased proliferation and their descendants undergoing accelerated differentiation.

As a higher-resolution approach, we used flow cytometry that allowed us to observe a significant decrease in the number of proliferative Ki-67^+^ cells in LPS-stimulated PC organoids compared to controls, indicating that LPS reduced epithelial proliferation (Fig. 5C). This conclusion was strengthened by observing concurrent downregulation in the expression of key genes of the Wnt pathway such as *Axin2* and *Ascl2* (Fig. 5D). These genes involved in Wnt signaling are expressed in the actively proliferating transit-amplifying compartment and repressed in the upper, noncycling, differentiated epithelial compartment (21, 22). These results were consistent with a lipid A-TLR4-mediated arrest of epithelial proliferation.

### *S. maltophilia* LPS induces programmed necroptosis in PC organoids.

**FIG 6:**
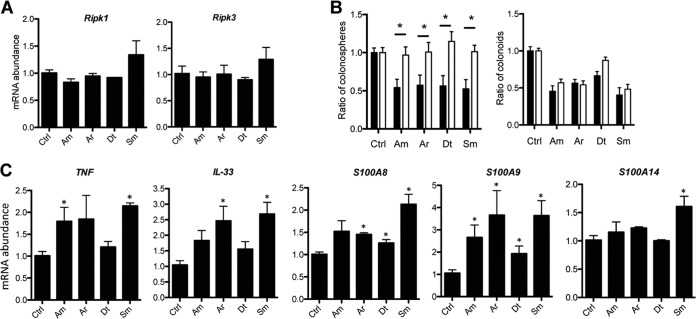
LPSs purified from CSCM members induce necroptosis and damage-associated molecular patterns (DAMPs) and IL-33. The CSCM members are *A. modestus* (Am), *A. radioresistens* (Ar), *D. tsuruhatensis* (Dt), and *S. maltophilia* (Sm). (A) Gene expression of *Ripk1* and *Ripk3* in LPS-stimulated organoids. (B) Ratio of colonospheres and colonoids during stimulation of organoids derived from WT mice (black bars) and RIPK3^−/−^ mice (white bars) with LPS purified from CSCM members (1.0 μg/ml). There were eight mice in each group. *, *P* < 0.05. (C) RT-PCR showing the expression of *TNF-α*, *IL-33*, *S100A8*, *S100A9*, and *S100A14*. There were six mice in each group indicated on the *x* axis. *, *P* < 0.05 versus nonstimulated control (Ctrl) group.

The TUNEL assay used in *in vivo* experiments did not allow us to discriminate between the two main forms of cell death, apoptosis and necroptosis (23, 24). In order to decipher the mechanisms by which LPS from CSCM species induced the death of PC organoids, we investigated the expression of apoptosis-associated proteins, such as cleaved caspase-3, cleaved caspase-8, caspase-9, p53, and PUMA (p53-upregulated modulator of apoptosis), and necroptosis-associated proteins, such as receptor-interacting protein kinases RIPK1 and RIPK3 in day 5 PC colonoids at gene and protein expression levels. Stimulation by *S. maltophilia* LPS reduced the expression of cleaved caspase-3 and PUMA, unlike *A. modestus* LPS, whereas stimulation by *A. radioresistens* and *D. tsuruhatensis* LPSs induced moderate reduction in the expression of cleaved caspase-3 (Fig. S5A). Under all conditions. p53 was barely detected. Gene transcription analysis of the *Bcl-2* and *Bcl-xL* genes that encode two antiapoptotic proteins indicated a moderate decrease in expression during stimulation of crypt cultures with *A. radioresistens* and *D. tsuruhatensis* LPS, whereas *S. maltophilia* LPS induced a decrease in expression of *Bcl-2*, but not of *Bcl-xL* (Fig. S5B). Only stimulation with *S. maltophilia* LPS induced an increase in the expression levels of the *Ripk1* and *Ripk3* genes (Fig. 6A), whereas at the protein level, stimulation with *A. modestus* LPS also upregulated RIPK1 and RIPK3 expression in colonoids at levels similar to that induced by *S. maltophilia* LPS (Fig. S5A). LPS from *A. radioresistens* and *D. tsuruhatensis* did not modify the expression of RIPK1 and RIPK3 (Fig. 6A). To validate the involvement of RIPK, we analyzed the ratios of PC colonospheres and colonoids derived from RIPK3^−/−^ mice. As shown in Fig. 6B, the set of purified LPSs from CSCM members did not induce a decrease in the number of colonospheres derived from the crypts of RIPK3^−/−^ mice, in contrast to what was observed in WT mice (Fig. 4), indicating that RIPK3 depletion could improve the survival rate in LPS-stimulated colonic crypt cultures (Fig. 6B). However, except when crypt cultures were grown in the presence of *D. tsuruhatensis* LPS, the others LPSs induced a decrease in the number of colonoids (Fig. 6B). As necroptosis is mediated by alpha tumor necrosis factor (TNF-α), crypt cultures were treated with a neutralizing anti-TNF-α LPS, resulting in incomplete, although significant, reversion of the deleterious effect of *S. maltophilia* LPS on colonospheres and colonoids (Fig. S6). On the basis of these results, we hypothesized that TNF-α-mediated necroptosis was preferentially induced in *S. maltophilia* LPS-stimulated organoids rather than apoptosis. Necroptosis not only involves the activation of death mediators such as RIPKs, but also the release of interleukin 33 (IL-33) and the products of damage-associated molecular pattern (DAMP) genes such as *S100A8*, *S100A9*, and *S100A14* (25). *S. maltophilia* LPS induced a significant increase in mRNA abundance of both IL-33 and the set of three DAMP-encoding genes in organoids. Stimulation with other LPS species caused intermediate levels of IL-33 expression. These three other LPS species also induced an increase in gene expression of *S100A9*, whereas the LPS from *A. modestus* did not modulate the expression of *S100A8* (Fig. 6C). These data were strongly supported by *in vivo* experiments showing upregulation of *Ripk1*, *IL-33*, and DAMP gene expression in the intestinal tissues of mice monocolonized with CSCM members (Fig. S7A). Taken together, these results demonstrate that LPS is the bacterial molecule causing necroptosis of epithelial cells, thereby leading to hypotrophic organoids.

10.1128/mBio.01680-17.6 FIG S5  LPS purified from CSCM members affect expression of apoptosis- and necroptosis-associated proteins in PC organoids. Protein lysates were prepared from day 5 PC organoids stimulated with LPS (1.0 μg/ml) or not stimulated with LPS. The CSCM members are *A. modestus* (Am), *A. radioresistens* (Ar), *D. tsuruhatensis* (Dt), and *S. maltophilia* (Sm). (A) Representative Western blot (left) and quantification (right) showing the expression of apoptosis-associated proteins (cleaved caspase-3 [C-casp3 and CC3], cleaved caspase-8 [C-casp8 and CC8], caspase-9 [Casp9 and C9], p53, and PUMA) and necroptosis-associated proteins (RIPK1 and RIPK3). Membranes were stripped and reprobed for actin. There were five mice in each group. (B) Gene expression analysis of two proapoptotic genes (*Bcl-2* and *Bcl-xL*) in LPS-stimulated organoids. There were four mice in each group. *, *P* < 0.05 versus nonstimulated control (Ctrl) group. Download FIG S5, JPG file, 0.2 MB. Copyright © 2017 Naito et al. 2017 Naito et al. This content is distributed under the terms of the Creative Commons Attribution 4.0 International license.

10.1128/mBio.01680-17.7 FIG S6  Anti-TNF antibody MP6-XT22 (10 μg/ml and 20 μg/ml) suppresses *S. maltophilia* LPS-induced PC organoid death. The ratio of PC colonospheres and colonoids was calculated on day 5. PC organoids were stimulated with *A. modestus* (Am) or *S. maltophilia* (Sm) LPS (1.0 μg/ml). There were six mice in each group. Error bars indicate standard errors of the means (SEM). *, *P* < 0.05 versus nonstimulated control (Ctrl) group. Download FIG S6, JPG file, 0.1 MB. Copyright © 2017 Naito et al. 2017 Naito et al. This content is distributed under the terms of the Creative Commons Attribution 4.0 International license.

10.1128/mBio.01680-17.8 FIG S7  Gene expression of genes involved in necroptosis in mice monocolonized with CSCM. The CSCM members are *A. modestus* (Am), *A. radioresistens* (Ar), *D. tsuruhatensis* (Dt), and *S. maltophilia* (Sm). (A) Gene expression analysis of genes involved in necroptosis during the time course of colonization. (B) Gene expression analysis of genes involved in tissue maturation 30 days after monocolonization. RNA was extracted from proximal colonic tissue of monocolonized mice. *, *P* < 0.05 versus nonstimulated control (Ctrl) group. Download FIG S7, JPG file, 0.1 MB. Copyright © 2017 Naito et al. 2017 Naito et al. This content is distributed under the terms of the Creative Commons Attribution 4.0 International license.

### LPS purified from CSCM-associated species stimulates the differentiation of epithelial cells in PC organoids.

**FIG 7:**
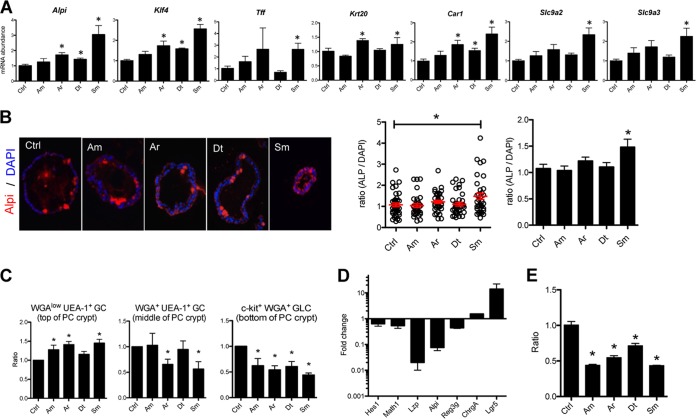
LPSs purified from CSCM members induce the differentiation/maturation of PC epithelial cells and act on Lgr5^+^ cells. (A) LPSs purified from CSCM strains induce mRNA abundance associated with maturated colonocyte transcripts. The CSCM members are *A. modestus* (Am), *A. radioresistens* (Ar), *D. tsuruhatensis* (Dt), and *S. maltophilia* (Sm). RT-PCR showing the expression of *Alpi*, *Klf4*, *Tff*, and keratin 20 (*krt20*) as mature epithelial cell markers and *Car1*, *Slc9a2*, and *Slc9a3* as markers of colonoids cultured from WT crypts. There were three mice in each group. *, *P* < 0.05 versus the nonstimulated control (Ctrl) group. (B) Representative micrographs of alkaline phosphatase (Alpi) immunostaining (red) of PC colonoids stimulated with LPSs from CSCM members. Nuclei were stained with 4′,6′-diamidino-2-phenylindole (DAPI) (blue). Quantitative analysis of the ratio of Alpi-stained area to DAPI-stained area were measured using the ImageJ software from day 5 PC colonoid stimulated with LPS (1.0 μg/ml). In the graph in the middle of panel B, the red bars indicate the average ratios of Alpi-positive area/DAPI-positive area of the PC organoid for the group. In the graph to the right in panel B, values are means plus standard errors (SE) (error bars). (C) Effects of LPSs from CSCM members on terminal differentiation of goblet cells (GC) in PC organoids. Fluorescence-activated cell sorting (FACS) analysis showing the ratios of WGA^+ ^UEA-1^+^ cells, which are located in the middle of the PC crypt, WGA-1^low ^UEA-1^+ ^cells, which are located at the top of the PC crypt, and c-kit^+ ^WGA^+^ goblet-like cells (GLC) in doublet and dead cells depleted of EpCam^+^ CD45.2^−^ cells from day 5 PC organoids. The organoids were from mice given *A. modestus* LPS (1.0 μg/ml), *A. radioresistens* LPS (1.0 μg/ml), *D. tsuruhatensis* LPS (1.0 μg/ml), or *S. maltophilia* LPS (1.0 μg/ml). There were six mice in each group. *, *P* < 0.05 versus nonstimulated control group. (D) Gene expression level of colonoids treated with CHIR99021 and valproic acid showing the upregulation of *Lgr5* expression and downregulation of genes involved in differentiation. (E) Ratio of PC colonoids during stimulation of organoids derived from C57BL/6 mice cultured with CHIR99021 and vaproic acid with LPSs from CSCM members (1.0 μg/ml).

Proliferation arrest is coupled with entry into a cell differentiation state. We investigated the possibility that the respective LPS may accelerate the terminal differentiation of PC epithelial cells into mature colonocytes. In order to address this issue, we combined transcriptional profiling by real-time PCR (RT-PCR) of a selection of representative genes and immunohistochemical staining. *S. maltophilia* LPS, compared to the three other LPSs, induced the expression of differentiation-associated genes signatures such as *Alpi*, *Klf4*, *Tff*, and *Krt20*, or colonocyte maturity markers such as carbonic anhydrase 1 (*Car1*) and solute carrier family 9/sodium/hydrogen exchangers 2 and 3 (*Slc9a2* and *Slc9a3*). Gene expression of Klf4, a zinc finger transcription factor regulating goblet cell differentiation in the colon was significantly upregulated by LPSs from *A. radioresistens*, *D. tsuruhatensis*, and *S. maltophilia* (Fig. 7A). In addition, upregulation of alkaline phosphatase was also demonstrated by immunohistochemistry (Fig. 7B). The influence of LPS on epithelial differentiation was also observed at a cell lineage level regarding goblet cells. An increased goblet cell ratio could be observed by flow cytometry, i.e., WGA^low ^UEA-1^+^ cells (WGA stands for wheat germ agglutinin, and UEA-1 stands for *Ulex europaeus* agglutinin 1) (26) (Fig. 7C) in agreement with the increased expression of *Klf4*. This increased expression of differentiation markers was not only the result of a decrease in global cell number and disappearance of nondifferentiated cells, because the PCR data were normalized to housekeeping genes of each sample, thus normalizing cell numbers.

Altogether, these results indicate that *S. maltophilia* LPS enhanced the differentiation of epithelial cells in PC organoids. Interestingly, in mice monocolonized with the four CSCM bacterial strains, we could also observe the upregulation of maturation markers, such as polymeric immunoglobulin receptor (*pIgr*) and mainly *Muc2* in the colonic epithelium (Fig. S7B).

In order to evaluate to which extent the LPS effect reflected direct targeting of stem cells, isolated crypts were cultured with CHIR99021, an inhibitor of the glycogen synthase kinase 3 (GSK-3), and valproic acid (VPA), a histone deacetylase (HDAC) inhibitor upon LPS stimulation. CHIR99021 and valproic acid are agents blocking the differentiation of intestinal cells and their combination induces an expansion of Lgr5-expressing stem cells (27). Compared to gene expression of colonoids in control medium, the presence of CHIR99021 and VPA induced an increase in Lgr5 expression and a decrease in expression of lysozyme (*Lzp*), *Alpi*, and *Reg3γ*, indicating strong enrichment in stem cells (Fig. 7D). Upon treatment of colonic crypt cultures with CHIR99021 and VPA, stimulation with the set of purified LPSs from CSCM members induced a decrease in organoids numbers, mainly composed of Lgr5^+^ stem cells (Fig. 7E). These data indicate that LPSs act directly on Lgr5^+^ intestinal stem cells in which TLR4 expression was detected (14) and thus participate in the regulation of homeostasis in a nonpathogenic context.

## DISCUSSION

The cross talk established between the gut microbiota and the intestinal epithelium is key to maintaining homeostasis in the gut. This robust symbiotic interaction likely stems from long coevolution under selective pressure for maintenance of vital physiological functions such as nutrition, barrier effect, and immune protection against intruding pathogenic microbes. Whether this coevolutionary process is a true mutualistic interaction that also benefits bacteria remains a central question. The mechanisms achieving robustness and resilience of the gut microbiota largely remain unclear, as well as the mechanisms of their disruption leading to a loss of balance, i.e., a dysbiosis, a new ecological state whose consequences may be neutral or possibly deleterious to the host (28, 29).

We recently decided to address these key issues by an approach establishing a “cellular microbiology of microbiota-host symbiosis.” For this, we needed to identify a well-defined anatomical, physiological, and microbiological niche to model and decipher relevant microbe-host molecular cross talks. We selected the intestinal crypt as such a niche, due to its basic function of epithelial regeneration. We hypothesized that the microbiota in general and possibly a “crypt-specific core microbiota (CSCM)” composed of a conserved/stable set of dedicated bacterial species, may be engaged in a symbiotic interaction to the benefit of optimizing epithelial regeneration. We gathered evidence for this, first demonstrating by sequencing 16S rRNA variable sequences the existence of a CSCM dwelling in the murine cecal and proximal colonic crypts (9). We also demonstrated the existence of a muramyl-dipeptide (MDP)–Nod2-mediated cytoprotective pathway of intestinal stem cells, thereby validating, at a molecular level, the principle of crypt-microbiota cross talk (10).

Renewal of the intestinal epithelium occurs every 5 days in humans and most mammals. Previous studies indicate that the microbiota or its metabolites play a role in cell turnover. Indeed, the intestinal epithelium of germfree mice is less developed than that of conventional mice (30), murine intestinal stem cells are protected against stress by an MDP-Nod2 pathway as previously mentioned (10), short-chain fatty acids modulated the cell cycle of intestinal epithelial cells (31), and it was very recently shown that butyrate delayed stem cell expansion in the crypt (32). The process of intestinal renewal bears largely upon Lgr5^+^ stem cells located at the bottom of intestinal crypts (33, 34). In the small intestine, activation of TLR4 reduces proliferation and induces apoptosis of epithelial cells through the PUMA pathway (14), through caspase-8 (35), or through endoplasmic reticulum (ER) stress (36).

Little is known regarding the colon; hence, this important issue needed clarification. As stated above, we previously demonstrated that a CSCM composed of a limited number of bacterial genera, mainly strictly aerobic and nonfermentative Gram-negative bacteria, inhabits the crypts of the murine proximal colon and cecum. In the reported work, we could confirm molecular identification data by cultivating species strictly corresponding to previously identified operational taxonomic units (OTUs) (*Acinetobacter*, *Delftia*, and *Stenotrophomonas*). In addition, to eliminate any breeding bias, we confirmed that *Acinetobacter*, the dominant CSCM genus, was also heavily present in the cecal and colonic crypts of wild rodents.

We hypothesized that CSCM members, due to close physical proximity, affected the physiology of the cecal and colonic regenerative apparatus, eventually maintaining its homeostasis (i.e., essentially proliferative rate but also inflammatory status) in the presence of microbiological, chemical, or physical aggressions. The steep oxygen gradient existing from the lamina propria to the intestinal lumen likely allows these aerobic species to live here (37, 38). In order to decipher and make sense of the cross talks established between these bacteria and the intestinal crypts, germfree mice were monocolonized with isolated CSCM strains, leading to a decrease in the proliferative rate and an increase in the death rate of colonic epithelial cells. This effect was not observed in germfree mice monoassociated with *B. fragilis*, a commensal Gram-negative bacterium that was not identified by 16S rRNA metataxonomics as a crypt-associated microbe in conventional mice, even if in monocolonized mice, *B. fragilis* could colonize the colonic crypts (15). Even if *B. fragilis* lipid A is penta-acylated and monophosphorylated in contrast to canonical biphosphorylated and hexa-acetylated enterobacterial lipid A (39), it was reported to be an inducer of alpha tumor necrosis factor (TNF-α) and IL-6 in human monocytes as well as IL-8 in TLR4/CD14/MD2-transfected HEK-293 cells (40), indicating its proinflammatory activity. The decrease in the proliferative rate and the increase in the death rate were also observed after stimulation of colonic crypts with sonicates and purified LPSs from CSCM members. This was not observed in mice deficient in TLR4, indicating the central role of LPS. These data are not consistent with a recent study showing that, in contrast to poly(I ⋅ C), LPS did not decrease colonoid viability (41). Other studies, however, showed that LPS reduced cell proliferation of the small intestinal epithelium (14, 42) and that TLR4 activation impaired enterocyte proliferation in the ilea of newborn mice (43). It was also shown that interferon (IFN) signaling could affect intestinal epithelial regeneration. Indeed, proliferative bromodeoxyuridine (BrdU)-positive cells increase in type I IFN receptor knockout mice, and it was demonstrated that this increase depends on microbiota composition (44). It was also shown that type I IFN controls the proliferation of the intestinal epithelium through its activation of β-catenin (45). In contrast to what was found in the small intestine (14), we showed that LPS-stimulated crypt death was caused by TNF-α-mediated necroptosis via the TNF receptor (TNFR)/RIPK1/RIPK3 pathway, rather than by apoptosis through the p53/PUMA pathway. Indeed, the deletion of RIPK3 improved the survival rate of colonic crypt cultures during stimulation with LPSs purified from CSCM members. Interestingly, it was recently shown that lung injury observed upon high-dose-LPS-induced acute respiratory distress syndrome in mice was due to RIPK3-mediated necroptosis (46). This could be linked to a study showing that sepsis affects quiescent muscle stem cells, causing a defect in muscle regeneration by inducing increased apoptosis of satellite cells (47). Necroptosis is induced by extracellular stimuli, first by TNF, and is mediated by RIPK3 via TNF receptor. RIPK3 is a downstream target of RIPK1, and the complex containing RIPK1 and RIPK3 is involved in initiating necroptosis (48–50). Other molecules involved in necroptosis, such as IL-33 and damage-associated molecular patterns (DAMPs) such as S100A8, S100A9, and S100A14 were also increased in LPS-stimulated crypt cultures. Differences observed following stimulation with different LPSs purified from CSCM members are likely to respond to structural differences of their corresponding lipid A. For instance, *D. tsuruhatensis* LPS that appeared the least active showed underacylation of its lipid A with very short fatty acid chains, a characteristic associated with a weak endotoxin activity (19, 20, 51). The association of endotoxins of different potencies may thus result in optimal endotoxic balance achieving the exact regulatory function on the crypt regenerative functions by regulating the life-to-death balance in the stem cell population.

We also showed that LPSs purified from CSCM members induced epithelial maturation toward the goblet cell lineage. It has been shown that the zinc finger transcription factor Klf4 is required for the differentiation of colonic goblet cells (52) and is induced during mucosal inflammation in Crohn’s disease. Here we show that LPSs from CSCM, mainly *S. maltophilia* LPS, which is hexa-acylated with highly heterogeneous fatty acid substitutions, induced the expression of *klf4* in colonoids and also increased the levels of goblet cells. In line with the upregulation of IL-33 expression and the increase in goblet cells by LPSs purified from CSCM, it was recently reported that IL-33 led to an expansion of goblet cells (53). In accordance with our results, a study indicates that, using human enteroids derived from the transverse colon, LPS induced an increase in goblet cell-associated proteins such as MUC2 (54). Altogether, these data indicate clearly that LPS impacts intestinal development by modulating stem cell activity.

LPS is the major cell wall component of Gram-negative bacteria and is released by shedding or through bacterial lysis (55). Indeed, it has been estimated that the amount of LPS released by Gram-negative bacteria could reach 50 μg/ml in the human colon (56). The role of LPS within the gut was so far essentially studied in pathogenic conditions, such as necrotizing enterocolitis, Crohn’s disease, and ulcerative colitis (57, 58). In these highly inflammatory conditions, circulating LPS levels are 40 to 60% higher in patients than in healthy subjects, likely reflecting increased luminal content and intestinal permeability, and suggesting a true pathogenic role of endotoxin (12). It was accordingly shown that TLR4 activation increased enterocyte apoptosis (59) and that activation of TLR4 by LPS inhibits enterocyte proliferation both *in vitro* on IEC-6 cell line and also *in vivo* (43). It was also shown that muramyl-dipeptide (MDP), the ligand of NOD2 was able to reverse the effects of LPS on the induction of enterocyte apoptosis (60). We already mentioned above that the addition of MDP to purified intestinal crypt caused an increase in organoid yield, mainly in cytotoxic stress conditions, by interacting directly with NOD2 located in Lgr5^+^ stem cells (10), indicating that different MAMPs could produce opposite effects on stem cells: a cytoprotective effect for the MDP-NOD2 interaction and necroptosis for the LPS-TLR4 interaction. Balancing these two pathways and the respective agonist potential of MAMPs from CSCM members on Lgr5^+^ stem cells, and further on progenitor and proliferating cells, may thus be essential to maintain intestinal crypt homeostasis. We hypothesize that this stable ecosystem isolates and protects the cecal and colonic crypts from brisk luminal variations in the concentrations of MAMPs and bacterial metabolites.

## MATERIALS AND METHODS

### Isolation of crypt-specific core microbiota from murine proximal colon.

Proximal colons from C57BL/6 mice (Elevage Janvier) were washed with bleach and homogenized in 2 ml of sterile phosphate-buffered saline (PBS) using the Precellys system with 2.8-mm ceramic beads. This mixture was then added to 30 ml of a minimum medium (0.04 M KH_2_PO_4_-Na_2_HPO_4 _[pH 6], 20 mM KNO_3_, 0.8 mM MgSO_4_ ⋅ 7H_2_O) with 14 mM sodium acetate ⋅ 3H_2_O as the carbon source (61). The cultures were incubated at 30°C for 48 h with shaking at 300 rpm/min in a Multitron incubation shaker (Infors). The cultures were then isolated on agar plates (GTCS, MacConkey, Herellea, and Chromagar). Selected colonies were reisolated on Chromagar to ensure that a pure colony was obtained. The colonies were identified using the Biolog system (GEN III microplate for both Gram-negative and Gram-positive bacteria; Biolog, Inc., Hayward, CA, USA). The identification of *Acinetobacter*, *Delftia*, and *Stenotrophomonas* was confirmed by Sanger sequencing of 16S rRNA, *rpoB*, and *gyrB* genes after genomic DNA extraction by the Wizard genomic DNA purification kit following the manufacturer’s instructions (Promega). The primers used are listed in Table S1 in the supplemental material.

10.1128/mBio.01680-17.9 TABLE S1  Sequences of primers for quantitative PCR. Download TABLE S1, DOCX file, 0.1 MB. Copyright © 2017 Naito et al. 2017 Naito et al. This content is distributed under the terms of the Creative Commons Attribution 4.0 International license.

### Extraction and analysis of LPS from CSCM strains.

Lipopolysaccharides (LPSs) from crypt-specific core microbiota (CSCM) strains were extracted as described previously (16). Briefly, each LPS was extracted from freeze-dried bacterial cell walls by the phenol-water method. The phase partitioning was repeated twice, and the water phase containing LPS was lyophilized. To further purify the LPS, RNase and DNase (Roche) were added (50 μg of each enzyme to 0.5 g of LPS in 20 ml of distilled water), and the solution was incubated at 37°C for 2 h. An equal volume of 90% phenol was added to denature the enzyme and possible adhering proteins. The solution was vortexed and centrifuged at 12,000 × *g* for 15 min. The upper water layer was removed, dialyzed against distilled water with three changes of water, and lyophilized. No protein was detected in the final preparation. In order to obtain lipid A, the LPSs were hydrolyzed with aqueous 1% acetic acid (AcOH) for 2 h at 100°C and centrifuged (11,000 rpm, 4°C, 1 h). In order to establish lipid A structure, the precipitate thus obtained underwent chemical analyses (17) and mass spectrometry (MS) (62).

10.1128/mBio.01680-17.1 TEXT S1 Supplemental Experimental Procedures and references. Download TEXT S1, PDF file, 0.2 MB. Copyright © 2017 Naito et al. 2017 Naito et al. This content is distributed under the terms of the Creative Commons Attribution 4.0 International license.
