# Supplementary figures and images for: Lipopolysaccharide from Crypt-Specific Core Microbiota Modulates the Colonic Epithelial Proliferation-to-Differentiation Balance
Source: mBio. 2017 Oct 17;8(5):e01680-17. doi: 10.1128/mBio.01680-17 (PMC5646255; doi:10.1128/mBio.01680-17)

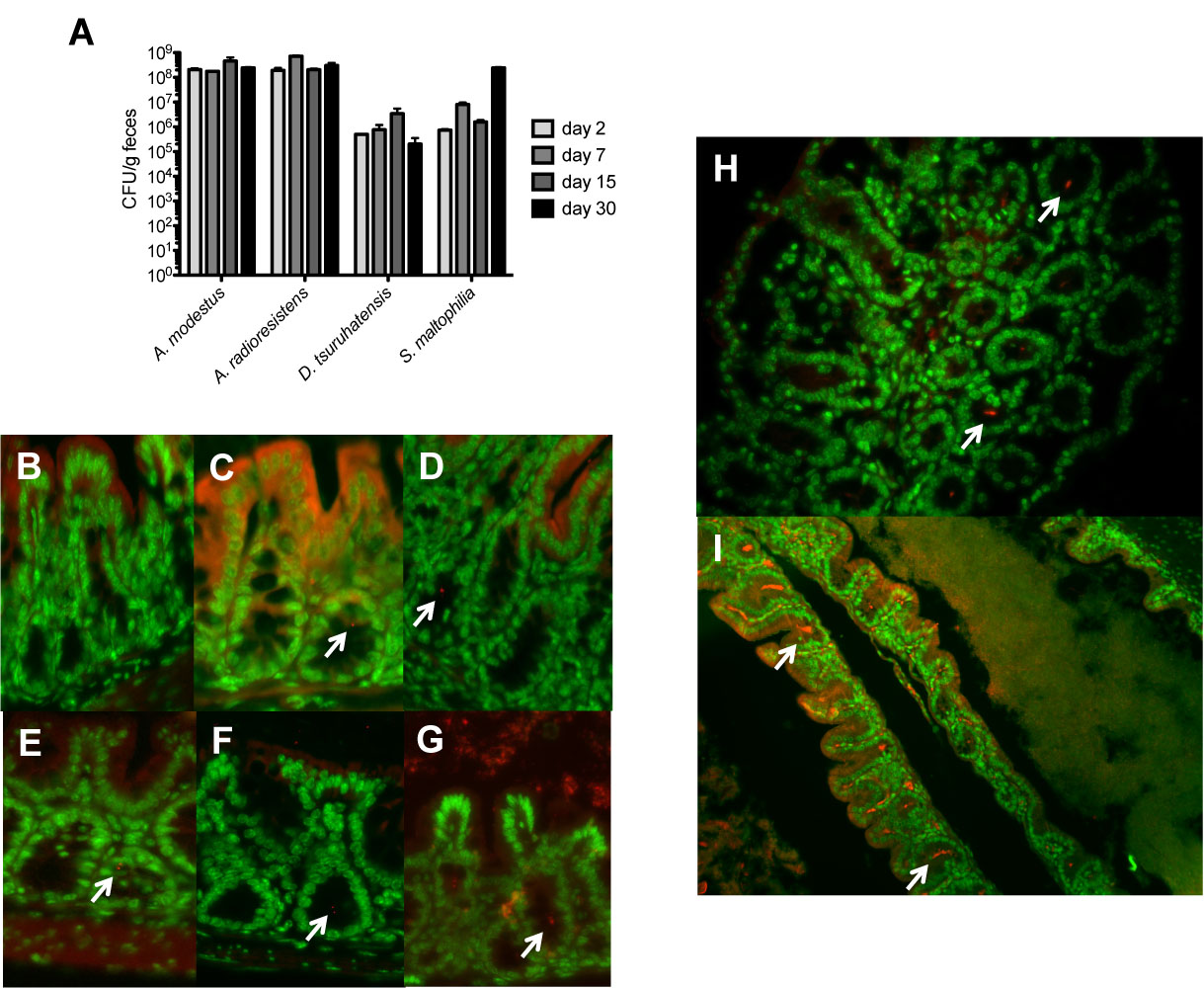

Supplement: FIG S1 [file mbo005173538sf1.jpg]

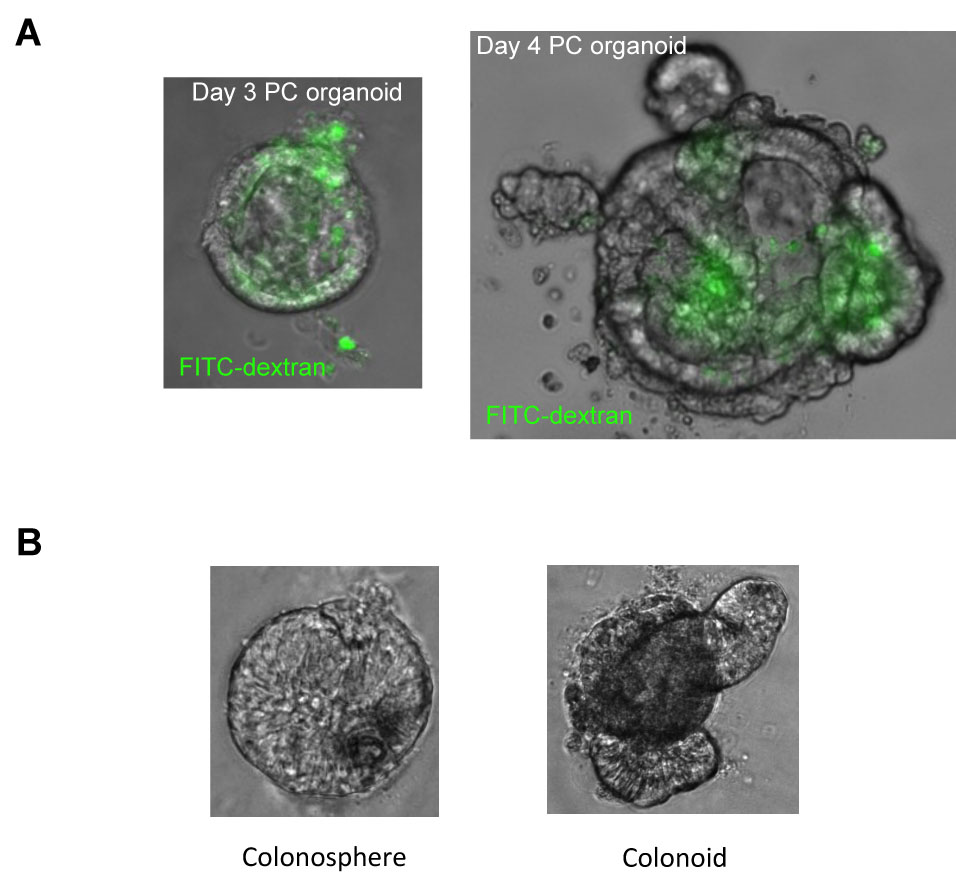

Supplement: FIG S2 [file mbo005173538sf2.jpg]

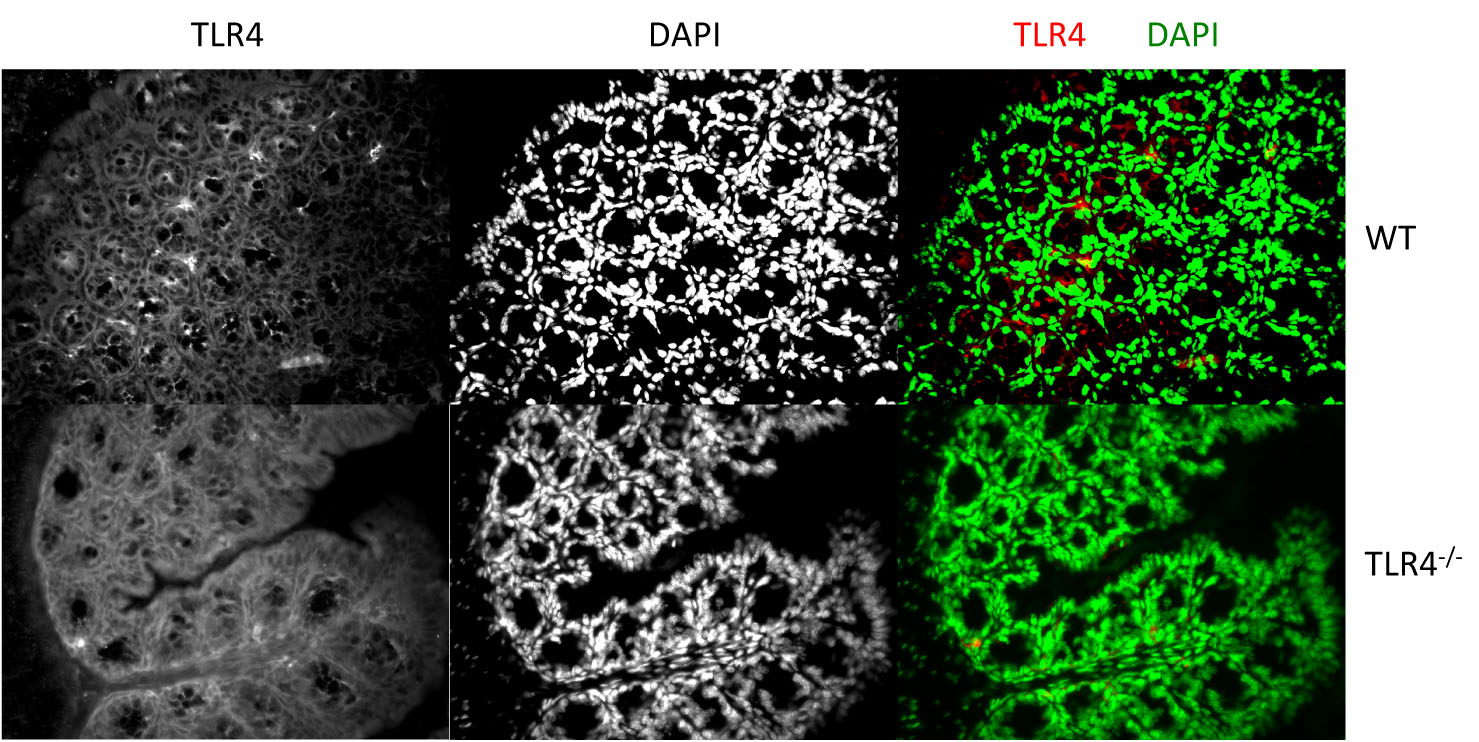

Supplement: FIG S3 [file mbo005173538sf3.jpg]

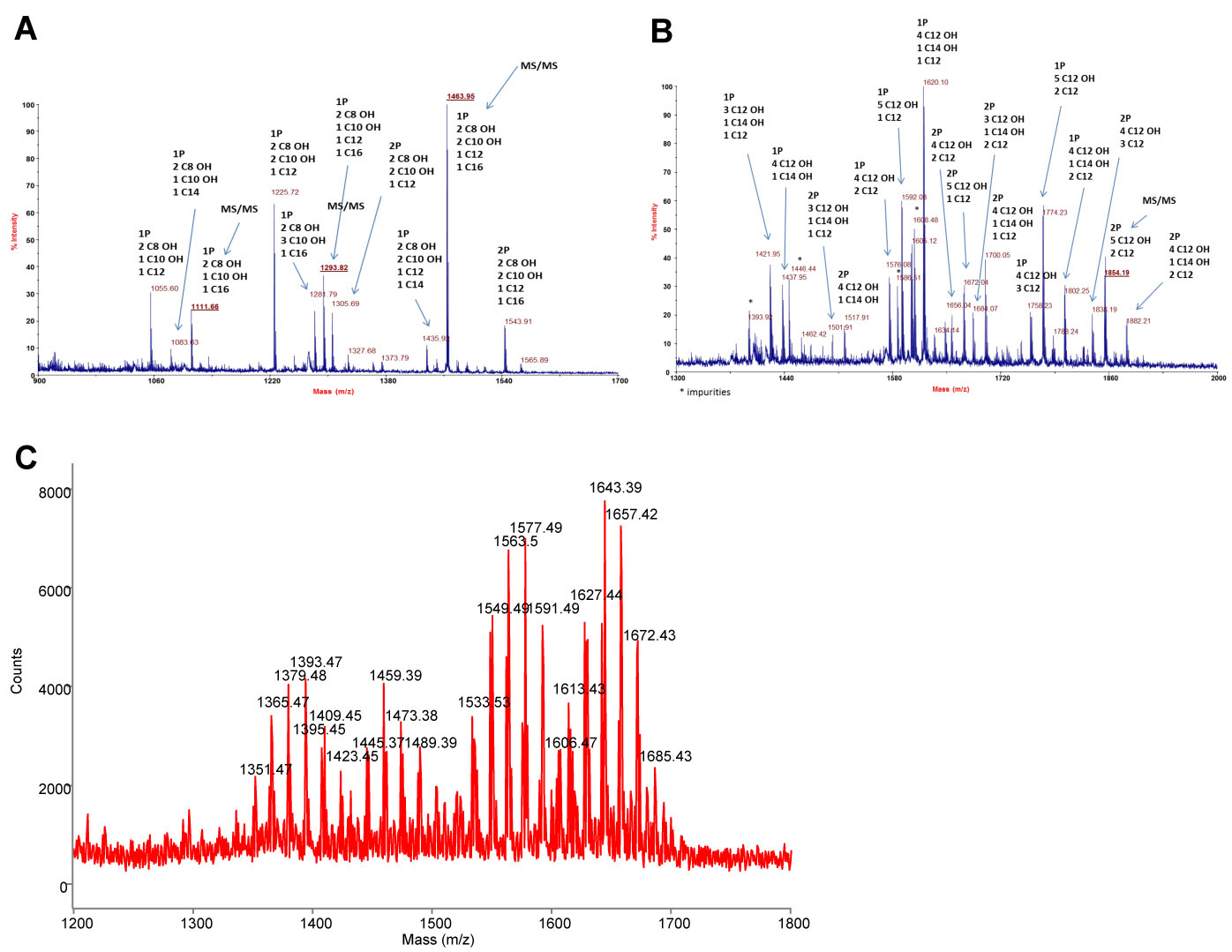

Supplement: FIG S4 [file mbo005173538sf4.jpg]

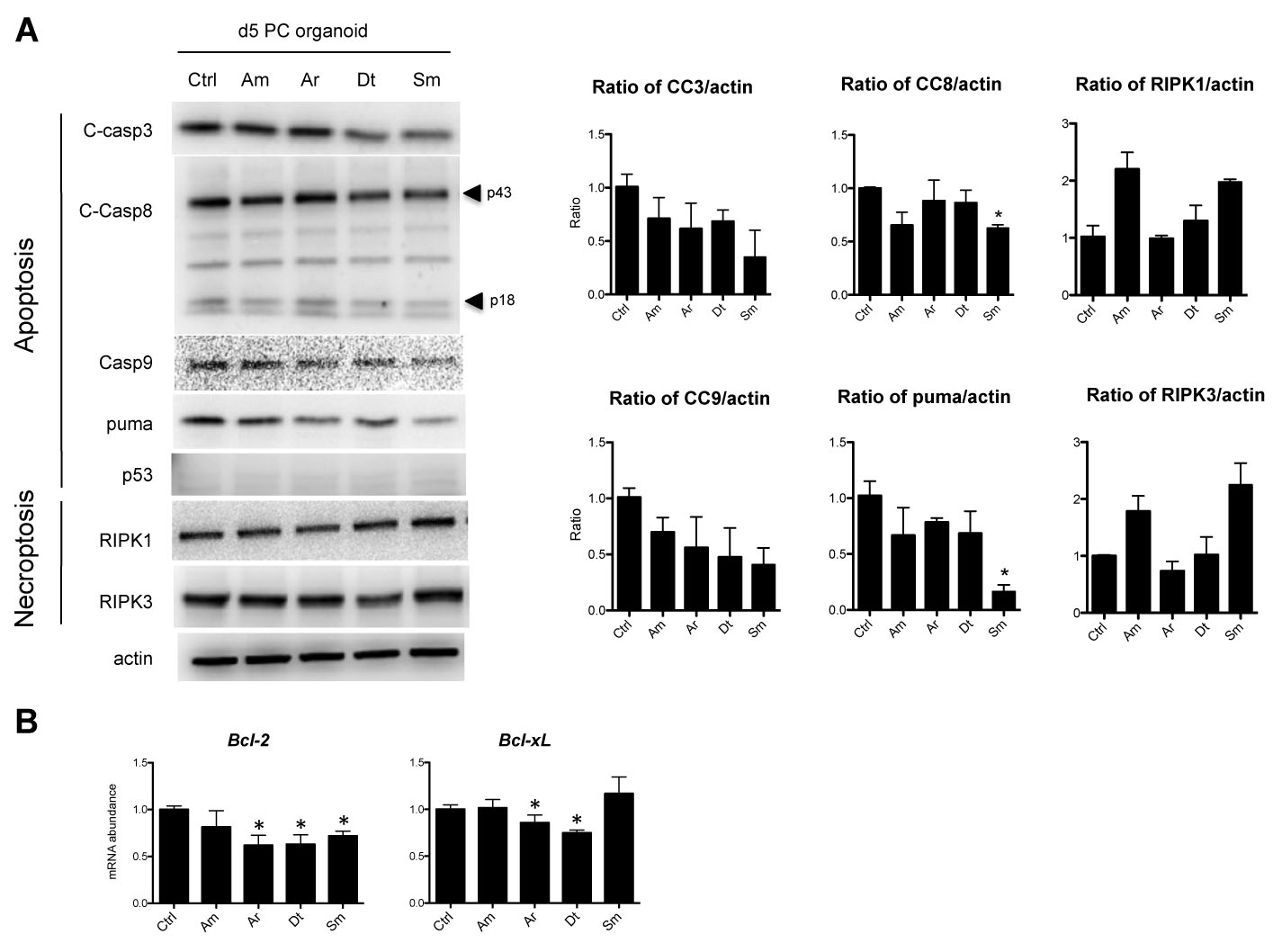

Supplement: FIG S5 [file mbo005173538sf5.jpg]

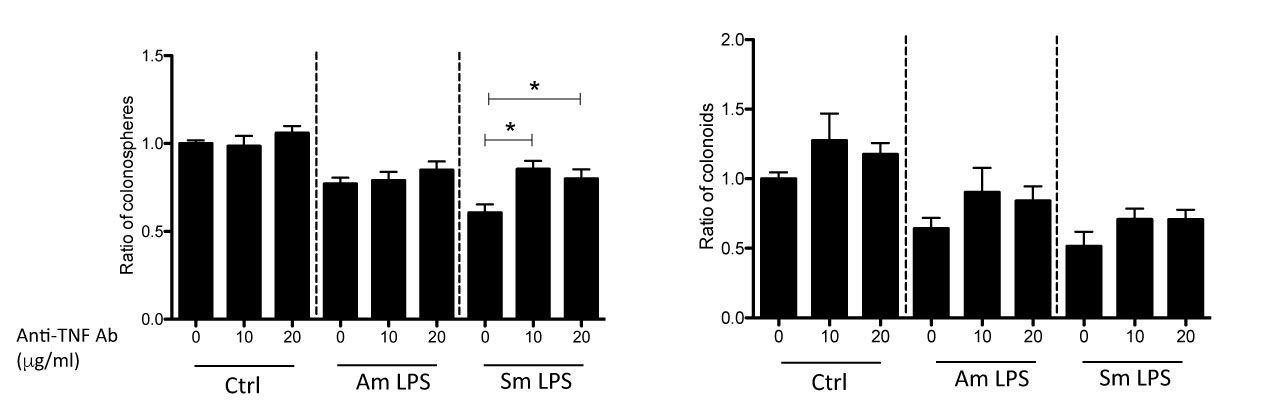

Supplement: FIG S6 [file mbo005173538sf6.jpg]

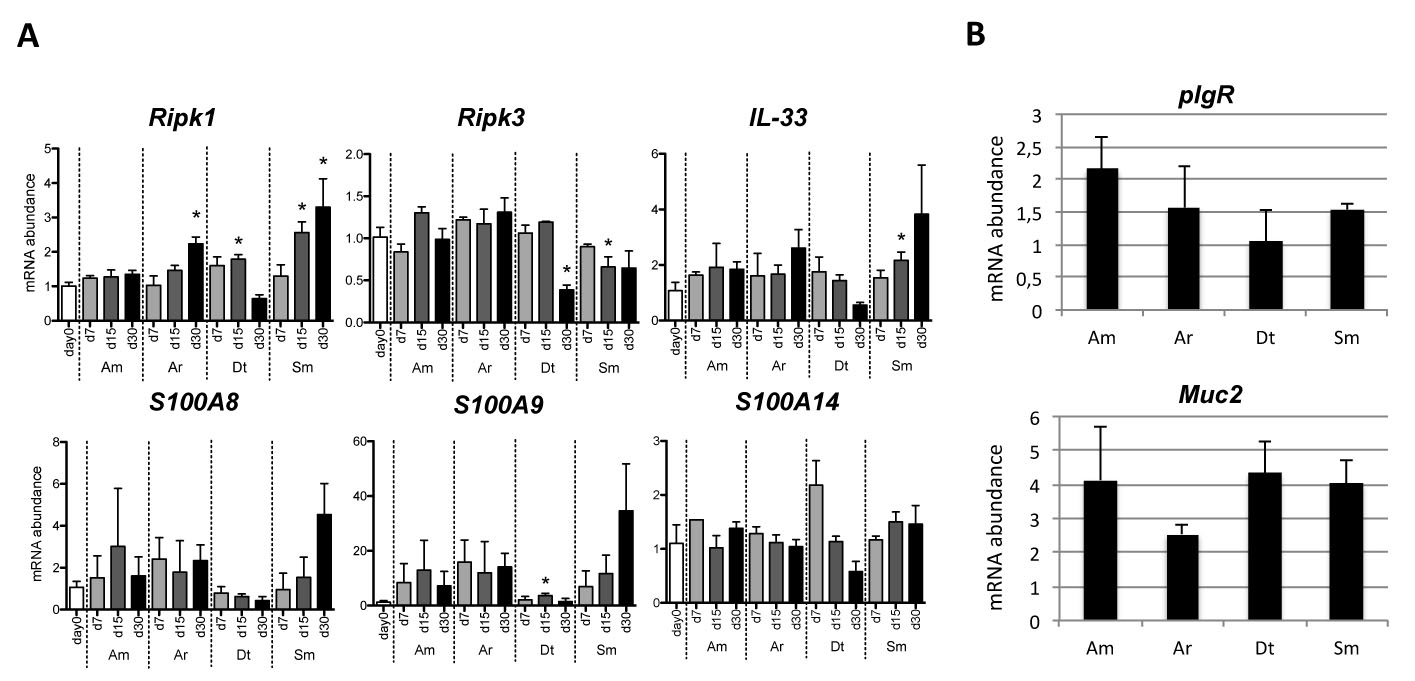

Supplement: FIG S7 [file mbo005173538sf7.jpg]
