# Supplementary material for: Lipopolysaccharide from Crypt-Specific Core Microbiota Modulates the Colonic Epithelial Proliferation-to-Differentiation Balance
Source: mBio. 2017 Oct 17;8(5):e01680-17. doi: 10.1128/mBio.01680-17 (PMC5646255; doi:10.1128/mBio.01680-17)
Supplement: TABLE S1 [file mbo005173538st1.docx]

**Supplemental Table S1**

| **Gene name** | **Forward primer** | **Reverse primer** | **Reference** |
| --- | --- | --- | --- |
| *Alpi* | TGGCTCTGTCCAAGACATACAG | CCGATGGTCTTGTAGTTGGTCT | 31 |
| *Ascl2* | AAGCACACCTTGACTGGTACG | AAGTGGACGTTTGCACCTTCA | S1 |
| *Axin2* | TGACTCTCCTTCCAGATCCCA | TGCCCACACTAGGCTGACA | S2 |
| *Car1* | GACAGTAGCAACCAATC | TTCATCAAAACGCCAAG | S3 |
| *ChrgA* | CACAGCCACCAATACC | TCTTCCTCCTCCTCTTC | S3 |
| *Gapdh* | CTTATCAGGCCAAGTATGATG | CAACCTGGTCCTCAGTGTAGC | 31 |
| *Hes1* | AGAGGCGAAGGGCAAGAATA | CGGAGGTGCTTCACAGTCAT | S4 |
| *IL-33* | GATGGGAAGAAGCTGATGGTG | TTGTGAAGGACGAAGAAGGC | S5 |
| *Klf4* | AGAGGAGCCCAAGCCAAAGAGG | CCACAGCCGTCCCAGTCACAGT | S4 |
| *krt20* | GGATTCGAGGTTCAAGTCACGG | TCTAGGTTGCGCTCCAGAGACT | S6 |
| *Lgr5* | CCTACTCGAAGACTTACCCAGT | GCATTGGGGTGAATGATAGCA | S1 |
| *LyzP* | GAGACCGAAGCACCGACTATG | CGGTTTTGACATTGTGTTCGC | S7 |
| *Math1* | AGAGACCTTCCCGTCTACCC | CTGCAAAGTGGGAGTCAGC | S4 |
| *Muc2* | GTCCGAAGTGTTACCCTGGAAT | CAGCTCTCGATGTGTGTGTAGGT | S8 |
| *pIgR* | CTCTTGGCCTACGATCTGTCTT | ACACCACCATGCTCCTTTAACT | 31 |
| *Reg3γ* | CCATCTTCACGTAGCAGC | CAAGATGTCCTGAGGGC | S9 |
| *Ripk1* | GGTCAAATTCAGAACAACCTGGA | CACACTGCGATCATTCTCGT | S10 |
| *Ripk3* | GAGATGGAAGACACGGCACT | GGTGGTGCTACCAAGGAGTT | S11 |
| *S100A14* | ATGGGACAGTGTCGGTCAG | GTGTCTCAATGGCCCTCTCT | S12 |
| *S100A8* | TGCGATGGTGATAAAAGTGG | GGCCAGAAGCTCTGCTACTC | S12 |
| *S100A9* | CACCCTGAGCAAGAAGGAAT | TGTCATTTATGAGGGCTTCATTT | S12 |
| *Slc9a2* | AGGAAAGTCGGTTGCC | GTCTACGGTCTGGATGG | S3 |
| *Slc9a3* | TGGCGTGGATTGTGTGAAAG | CAGCAGGAAGGCGAAGATAAC | S3 |
| *Tff3* | AGTGGTCCTGAAGC | CGATGTGACAGAAGG | S3 |
| *TNFα* | CCAGACCCTCACACTCA | CACTTGGTGGTTTGCTACGAC | S13 |
| *16S rDNA* | AGAGTTTGATCCTGGCTCAG | GACGGGCGGTGWGTRCA | S14 |
| *GyrB* | GAAGTCATCATGACCGTTCTGCA | AGCAGGGTACGGATGTGCGAGCC | S14 |
| *RpoB* | TAYCGYAAAGAYTTGAAAGAAG | CGBGCRTGCATYTTGTCRT | S15 |
