# Supplementary material for: Lipopolysaccharide from Crypt-Specific Core Microbiota Modulates the Colonic Epithelial Proliferation-to-Differentiation Balance
Source: mBio. 2017 Oct 17;8(5):e01680-17. doi: 10.1128/mBio.01680-17 (PMC5646255; doi:10.1128/mBio.01680-17)
Supplement: TEXT S1 [file mbo005173538s1.pdf]

Supplemental Experimental Procedures

Mice

Six- to ten- week old C57BL/6 mice from Elevage Janvier were used in this study. C57BL/6 germ-free mice were from the Institut Pasteur animal facilities. Toll like receptor 4 knockout and RIPK3 knockout mice were provided respectively by S. Akira and V. Dixit. Animals were housed in the Institut Pasteur animal facility accredited by the French Ministry of Agriculture for performing experiments on live rodents. Work on animals was performed in compliance with French and European regulations on care and protection of laboratory animals (EC Directive 2010/63, French Law 2013-118, February 6th, 2013). All experiments were approved by the Ethics Committee #89 and registered under the reference 2013-0030. Wood mice and bank voles were trapped as described (S16). From the 30th September - 1st October 2015, wild rodents were trapped on three 1 ha grids at Callendar Wood, Falkirk, UK (55°59' N, 3°46' W). Live traps (H.B. Sherman 2 x 2.5 x 6.5 inch folding traps, Tallahassee, FL, USA) were set at dusk (2 traps every 10m, 300 traps total) and baited with bedding, mixed seeds, mealworms and carrot. The following morning all traps were checked and five wood mice (*Apodemus sylvaticus*) and five bank voles (*Myodes glareolus*) were sacrificed. Their complete intestine from duodenum up to and including colon was removed in the field and fixed immediately in 4% Paraformaldehyde (PFA). After 7 days at 4°C, intestines were transferred from PFA to 70% ethanol for storage.

Selection of streptomycin resistant bacteria.

A. radioresistens CM38.2 isolated from murine proximal colonic crypt was grown overnight in Trypticase Soy Broth. After centrifugation the pellet containing 10^{11} CFU was platted on Trypticase Soy Agar containing streptomycin (100 µg/ml). Two days later seven colonies were obtained and re-isolated on streptomycin–agar plates. The day after one colony was selected for *in vivo* experiment.

Sonicated bacteria

Overnight cultures of bacterial strains cultured in Trypticase-Casein-Soy Broth were adjusted at 5.0 x 10^8 cells/ml. Samples were centrifuged at 10,000 g for 5 min and re-suspended at the same concentration in saline solution. Samples were then sonicated 3 times during 10 s using ultrasonic homogenizer (UP50H, Hielscher). After a centrifugation at 12,000 g for 3 min, the supernatants were filtered twice through 0.22 µm and aliquots were stored at -20°C.

LPS detoxification

Deacylation of LPS was performed during a treatment for six hours at 37°C with 4M KOH followed by a neutralization with 4M HCL as described by O. Holst (S17).

Crypts isolation and organoids formation.

Intestinal crypts were extracted as previously described with minor modification (S18). Proximal colon was removed from mice and the content was flushed with PBS containing 3% bleach. Flushed colon was inside-out on a wood skewer and incubated into 10 mM EDTA cold chelation buffer (Distilled water with 5.6 mM Na_2HPO_4, 8.0 mM KH_2PO_4, 96.2 mM NaCl, 1.6 mM KCl, 43.6 mM sucrose, 54.9 mM D-sorbitol, 0.5 mM DL-dithiothreitol) for 30 min on ice. Skewer was transferred into new 10 mM EDTA chelation buffer and vigorously vortex mixed for 10 min, transferred in a new 10 mM EDTA chelation buffer and incubated for 15 min on ice. This step was repeated 4-5 times. The supernatant containing crypts were filtered by 100 µm mesh, collected into 50 ml falcon tube and centrifuged at 150-200 g for 5 min. Proximal colonic crypts were pelleted and counted under light microscopy. 500 crypts were embedded in 50 µl matrigel (growth factor reduced, BD Bioscience) and plated in 24 wells plate, polymerized for 10-15 min at 37 °C and 500 µl/well basal culture media (advanced Dulbecco's modified Eagle medium/F12 supplemented with 100 U/ml penicillin 100 µg/ml streptomycin, 10 mM Hepes, 1x Glutamax, 1 x N2, 1 x B27 and 1 mM N-acetylcysteine) were added containing the following growth factor combination: 10% FBS, 50 ng/ml murine EGF (R&D), 500 ng/ml human R-spondin (R&D), 100 ng/ml murine wnt3A (R&D), 25 ng/ml murine Noggin (PeproTech) and 50 ng/ml murine HGF (R&D). Medium was changed every 2 days.

Flow cytometric analysis of PC organoids

Matrigel was removed and cultured organoids were collected using BD Cell Recovery Solution (Corning). PC organoids were dissociated by TrypLE Express (Thermo Fisher) for 40 min at 37 °C. Dissociated cells were passed through 40 µm cell strainer (BD) and washed by HBSS containing with 2% FBS. Viable single cells were gated by forward scatter, side scatter, pulse width to remove doublet cells, negative staining for zombie aqua (Bio Legend) to eliminate dead cells using FACS Canto (BD). Epithelial cells were gated using an anti-Epcam (positive selection) and an anti-CD45 (negative selection) and the data were analyzed by FlowJo software.

RNA extraction

Collected PC organoid were washed by PBS and lysed following the manufacture's protocol using total RNA isolation micro kit (Macherey Nagel). One cm of proximal colonic tissue was homogenized in 2 ml tubes containing 0.1 mm glass beads with 1 ml Trizol using the Precellys system. After extraction with chloroform, precipitation with isopropanol and washings with 70% ethanol, the extracted RNA was resuspended in 100 µl of water. A clean-up of the RNA was performed with the Nucleospin RNA II kit (Macherey-Nagel). RNA quantification was done with the NanoDrop ND-100 (Thermo Scientific). cDNA synthesis was performed from 1 µg (for the organoid) or 2 µg (for intestinal tissue) of RNA using oligo-dT (Promega) and Superscript II (Life Technologies).

Real time PCR (RT-PCR).

RT-PCRs were carried out in a 15 µl volume containing 6 µl of cDNA (at 2 µg/ml), specific primers (0.2 µM, listed in supplementary Table S1), and 7.5 µl of FastStart Universal SYBR Green Master (Roche). Reactions were run on a QuantStudio 7 (Applied Biosystems) according to the manufacturer's instructions. Each reaction was run in duplicate. Glyceraldehyde-3-phosphate dehydrogenase (GAPDH) RNA was used as an internal control gene, and ΔΔ Ct (cycle threshold) values were calculated to obtain relative expression. Means and SD were calculated at least from three experiments, and the values were analyzed by Mann-Whitney U test.

Fluorescence *in situ* hybridization (FISH)

Intestinal tissues were fixed overnight at 4°C in 4% paraformaldehyde in PBS and processed for paraffin embedding. Thin section of 7 µm were cut, dried, and stored at 4°C. Sections were dewaxed, re-hydrated and covered with a solution of lysozyme at 10 mg/ml in PBS during 20 min at 37°C, and washed twice with PBS. After 30 min of incubation in hybridization buffer (20 mM Tris-HCl [pH 8], 0.9 M NaCl, 0.01% SDS, 30% formamide), slides were incubated overnight in hybridization buffer containing 50 nM of the fluorescent probes at 42°C. After washing twice in 1× SSC (0.15 M NaCl, 0.015 M sodium citrate), slides were covered for 20 s with 4,6-diamidino-2-phenylindole (DAPI) (0.125 µg/ml in PBS), washed in PBS, and mounted in ProLong gold antifade reagent (Thermo Fisher). The 16S rRNA-targeted oligonucleotide probe Eub338 (GCTGCCTCCCGTAGGAGT) or *Acinetobacter* specific probe ACA652 (ATCCTCTCCCATACTCTA) were covalently linked with Alexa 555 at their 5' end (S19). Slides were examined under an Olympus IX81 microscope equipped with a CCD camera, and images were processed using the MetaVue software program or under a Widefield ApoTome inverted microscope (Zeiss) using the Axovision software program.

Antibodies and lectins used for FACS analysis and immunohistochemistry

The followings antibodies and lectins used for FACS analysis and immunohistochemical analysis were: Alexa 488 conjugated EpCAM (118210, Biolegend) or PE-Cy7 conjugated EpCAM (25-5791, eBioscience); APC conjugated CD45.2 (109813, Biolegend); PE conjugated-ki67 (652403, Biolegend); PE-Cy7 conjugated c-kit (105813, Biolegend) or Alexa488 conjugated c-kit (105815, Biolegend); PE-Cy7 conjugated TLR4 (117609, Biolegend) or LEAF purified TLR4 (117607, Biolegend); rabbit anti Muc2 (sc15334, Santacruz); goat anti-mouse Alkaline phosphatase (AF2910, R&D) ; FITC conjugated UEA1 (L9006, Sigma); Alexa 555 conjugated WGA (W32464, Sigma).

Immunofluorescence staining

Colonic tissue were dissected, embedded in OCT compound, accurately frozen and sliced at 6 μm using Cryostat (Leica). Sectioned tissue was fixed by 4% PFA, immunofluorescence staining was performed. Grown organoids were fixed by 4% PFA for 1h at 4°C, sequentially incubated in 10, 15 and 20% sucrose at 4°C. Fixed organoids were embedded in OCT compound, frozen and sliced at 6 µm thickness with standard techniques. Immunofluorescence staining was examined following by standard methods. Nuclei were counterstained with DAPI. Stained slides were observed under an Eclipse E800 microscope (Nikon). Image processing was carried out using Image J software. Quantification of alkaline phosphatase positive area and grown organoid area was performed using Image J.

Western blotting analysis

Collected PC organoid or crypts were suspended in lysis buffer containing 50 mM Tris-HCl, 0.15 M NaCl, 0.1% SDS, 1% Triton X-100, 1% Sodium deoxycholate, 1mM PMSF and 1 µg /ml aprotinin. Lysates were sonicated, centrifuged and supernatants collected. Proteins were separated by SDS-PAGE using precast gels (BioRad) and transferred to polyvinylidene difluoride membrane (Millipore). Membranes were blocked by TBST containing 3% BSA and incubated with the following primary antibodies: actin (A2066, Sigma); p53 (NCL-p53-CMp, Leica); Puma (ab54288, Abcam); RIPK1 (610459, BD); RIPK3 (ADI-905-242, Enzo); cleaved caspase-3 (9664, CST); cleaved caspase-8 (9429, CST); caspase-9 (9508, CST) followed by corresponding HRP conjugated anti-

rabbit IgG (GAR/IgG(H+L), Nordic Immunology) or HRP conjugated anti-mouse IgG (NXA931, GE healthcare). Specific proteins were detected by chemiluminescence (SuperSignal West Dura or Femto) (Thermo Fisher) using ChemiDocTM XRS+ system (BioRad).

***In vivo* mono-colonization**

Seven weeks old germ-free C57BL/6 mice were inoculated by gavage at day 0 and day 2 with 5 x 10^8 bacteria in 100 ml PBS. Colonization was followed by counting the CFU in the feces. Briefly, feces were homogenized in 7 ml precellys tubes with 2.8 mm beads in 2 ml PBS and serial dilutions were platted on trypticase-soy agar plates. The number of bacteria was normalized to 1 gram of stool (Figure S1A). The localization of the bacteria was visualized by FISH using the Eub338 as described above (Figure S1B-S1G). For histological analysis, one cm of duodenum, jejunum, ileum, proximal and distal colon and the all cecum were fixed in Carnoy solution (60% ethanol, 30% chloroform and 10% glacial acetic acid) in order to preserve the mucus layer. After dehydratation, tissues were embedded in paraffin and cut in 7 µm section with a microtome. Mitotic cells were analyzed by EdU staining. Two hours before sacrifice, mice received an i.p. injection of 0.2 mg of EdU in 100 ml of PBS. Revelation of EdU staining was done using Click-iT Edu Imaging kit (Thermo Fisher) following manufacture's instructions. Dead or apoptotic cells were detected using the TdT In Situ Apoptosis Detection Kit - DAB TUNEL-based Apoptosis Detection Assay (R&D). After Hematoxylin and Eosin staining, crypt length was determined by bottom-to-surface measurement of at least 30 correctly aligned crypts of three mice per group. Measurements were done with ImageJ software.

***In vivo* colonization of Specific Pathogen Free mice**

6- to 8-week old C57BL/6 male mice or $TLR4^{-/-}$ mice raised in specific pathogen free (SPF) conditions were given autoclaved drinking water containing streptomycin (5g/L) for one day. The animals were then gavaged with 100 µl of a suspension of 2 x 10^8 CFUs of Streptomycin resistant *A. radioresistens*. Streptomycin-treated mice gavaged with physiological water were used as controls. During the two weeks of the experiment the water containing streptomycin was let to the animals.

Mice fecal samples were collected at days 5, 8 and 15 and diluted in PBS, and serial dilutions were plated in Trypticase Soy agar supplemented with streptomycin (100 µg/ml). Plates were incubated overnight at 37°C.

At day 15 the mice received an i.p. injection of 0.2 mg of EdU in 100 µl of PBS. Two hours later the mice were sacrificed and proximal colonic tissues were recovered for RNA extraction in one ml of Trizol and histological analysis (EdU and Tunel assay) in 2 ml of PFA 4% before embedding in paraffin.

Supplemental References

S1. **Hirata A, Utikal J, Yamashita S, Aoki H, Watanabe A, Yamamoto T, Okano H, Bardeesy N, Kunisada T, Ushijima T, Hara A, Jaenisch R, Hochedlinger K, Yamada Y.** 2013. Dose-dependent roles for canonical Wnt signalling in de novo crypt formation and cell cycle properties of the colonic epithelium. Development **140**, 66-75.

S2. **Haung H, Cotton JL, Wang Y, Rajurkar M, Zhu LJ, Lewis BC, Mao J.** 2013. Specific requirement of Gli transcription factors in hedgehog-mediated intestinal development. J. Biol. Chem. **288**, 17589-175896.

S3. **Beuling E, Aronson BE, Tran LM, Stapleton KA, ter Horst EN, Vissers LA, Verzi MP, Krasinski SD.** 2012. GATA6 is required for proliferation, migration, secretory cell maturation, and gene expression in the mature mouse colon. Mol Cell Biol **32(17)**:3392-3402.

S4. **Becker S, Oelschlaeger, TA, Wullaert A, Vlantis K, Pasparakis M, Wehkamp J, Stange EF, Gersemann M.** 2013. Bacteria regulate intestinal epithelial cell differentiation factors both in vitro and in vivo. PLoS One **8(2)**:e55620.

S5. **Zhao WH, Hu ZQ**. 2012. Up-regulation of IL-33 expression in various types of murine cells by IL-3 and IL-4. Cytokine **58**:267-273.

S6. **Sommer F, Backhed F.** 2015. The gut microbiota engages different signaling pathways to induce Duox2 expression in the ileum and colon epithelium. Mucosal. Immunol. **8**:327-329.

S7. **Tang C, Kamiya T, Liu Y, Kadoki M, Kakuta S, Oshima K, Hattori M, Takeshita K, Kanai T, Saijo S, Ohno N, Iwakura Y.** (2015). Inhibition of Dectin-1 Signaling Ameliorates Colitis by Inducing Lactobacillus-Mediated Regulatory T Cell Expansion in the Intestine. Cell Host Microbe **18(2)**:183-197.

S8. **Buczacki SJ, Zecchini HI, Nicholson AM, Russell R, Vermeulen L, Kemp R, Winton DJ.** 2013. Intestinal label-retaining cells are secretory precursors expressing Lgr5. Nature **495(7439)**/65-69.

S9. **Burger-van Paassen N, Loonen LM, Witte-Bouma J, Korteland-van Male AM, de Bruijn AC, van der Sluis M, Lu P, Van Goudoever JB, Wells JM, Dekker J, Van Seuningen I, Renes IB.** 2012. Mucin Muc2 deficiency and weaning influences the expression of the innate defense genes Reg3, reg3γ and angiogenin-4. PLoS One **7**:e38798.

S10. **Takahashi N, Vereecke L, Bertrand MJ, Duprez L, Berger SB, Divert T, Gonçalves A, Sze M, Gilbert B, Kourula S, Goossens V, Lefebvre S, Günther C, Becker C, Bertin J, Gough PJ, Declercq W, van Loo G, Vandenabeele P.** (2014). RIPK1 ensures intestinal homeostasis by protecting the epithelium against apoptosis. Nature **513(7516)**:95-99.

S11. **Moriwaki K, Balaji S, McQuade T, Malhotra N, Kang J, Chan FK.** 2014. The necroptosis adaptor RIPK3 promotes injury-induced cytokine expression and tissue repair. Immunity **41(4)**:567-578.

S12. **Song X, Zhu S, Shi P, Liu Y, Shi Y, Levin SD, Qian Y.** 2011. IL-17RE is the functional receptor for IL-17C and mediates mucosal immunity to infection with intestinal pathogens. Nat Immunol **12**:1151-1158.

S13. **Reigstad CS, Lundén GO, Felin J, Bäckhed F.** 2009. Regulation of serum amyloid A3 (SAA3) in mouse colonic epithelium and adipose tissue by the intestinal microbiota. PLoS One. **4(6)**:e5842.

S14. **Pickard JM, Maurice CF, Kinnebrew MA, Abt MC, Schenten D, Golovkina TV, Bogatyrev SR, Ismagilov RF, Pamer EG, Turnbaugh PJ, Chervonsky AV.** 2014. Rapid fucosylation of intestinal epithelium sustains host-commensal symbiosis in sickness. Nature **514**:638-641.

S15. **Nemec A, Musílek M, Maixnerová M, De Baere T, van der Reijden TJ, Vaneechoutte M, Dijkshoorn L.** 2009. Acinetobacter beijerinckii sp. nov. and Acinetobacter gyllenbergii sp. nov., haemolytic organisms isolated from humans. Int J Syst Evol Microbiol **59**:118-124.

S16. **Maurice CF, Knowles SC, Ladau J, Pollard KS, Fenton A, Pedersen AB, Turnbaugh PJ.** 2015. Marked seasonal variation in the wild mouse gut microbiota. ISME J **9(11)**:2423-34.

S17. **Holst O.** 2000. Deacylation of lipopolysaccharides and isolation of oligosaccharide phosphates. Methods Mol Biol **145** :345-353.

S18. **Sato T, Stange DE, Ferrante M, Vries RG, Van Es JH, Van den Brink S, Van Houdt WJ, Pronk A, Van Gorp J, Siersema PD, Clevers H.** 2011. Long-term expansion of epithelial organoids from human colon, adenoma, adenocarcinoma, and Barrett's epithelium. Gastroenterology **141(5)**:1762-1772.

S19. **Greuter D**, **Loy A**, **Horn M**, **Rattei T**. 2016. probeBase--an online resource for rRNA-targeted oligonucleotide probes and primers: new features 2016. Nucleic Acids Res **44(D1)**:D586-589.
